# Mushroom tyrosinase enzyme catalysis: synthesis of larvicidal active geranylacetone derivatives against *Culex quinquesfasciatus* and molecular docking studies

**DOI:** 10.3389/fchem.2023.1303479

**Published:** 2024-01-10

**Authors:** Janani Mullaivendhan, Anis Ahamed, Ibrahim A. Arif, Gurusamy Raman, Idhayadhulla Akbar

**Affiliations:** ^1^ Research Department of Chemistry, Nehru Memorial College (Affiliated Bharathidasan University), Puthanampatti, Tamil Nadu, India; ^2^ Department of Botany and Microbiology, College of Science, King Saud University, Riyadh, Saudi Arabia; ^3^ Department of Life Science, Yeungnam University, Gyeongsan, Republic of Korea

**Keywords:** Mannich base, geranylacetone derivatives, larvicidal activity, ichthyotoxicity, molecular docking studies

## Abstract

The grindstone process, which uses tyrosinase as a catalyst, was used to create analogues of geranylacetone. Tyrosinase was used to prepare the Mannich base under favourable reaction conditions, resulting in a high yield. All synthesized compounds were characterized using FTIR, Nuclear magnetic resonance, and mass spectral analyses. The active geranylacetone derivatives (**1a-l**) were investigated for larvicidal activity against *Culex quinquefasciatus*; compound **1b** (LD_50_:20.7 μg/mL) was noticeably more effective than geranylacetone (LD_50_: >100 μg/mL) and permethrin (LD_50_: 24.4 μg/mL) lead compounds because of their ability to kill larvae and use them as pesticides. All compounds **(1a-1l)** were found to be low toxic, whereas compounds **1b, 1d**, and **1k** were screened for antifeedant screening of non -aquatic target for the toxicity measurement against marine fish *Oreochromis mossambicus* at 100 μg/mL caused 0% mortality in within 24 h. Molecular docking studies of synthesised compound **1b** and permethrin docked with 3OGN, compound **1b** demonstrated a greater binding affinity (−9.6 kcal/mol) compared to permethrin (−10.5 kcal/mol). According to these results, the newly synthesised geranylacetone derivatives can serve as lead molecules of larvicides agents.

## 1 Introduction

Mosquitoes play an important role in malaria transmission and have significant economic and social consequences worldwide. *Culex quinquefasciatus*, a type of mosquito, is linked to disease spread through vectors in several regions (Anoopkumar and Aneesh, 2022). Methoprene, a chemical found in some larvicides, prevents larvae from becoming adults (Wijayaratne et al., 2018). Researchers have studied botanical insecticides to identify alternatives to synthetic insecticides. Natural, biodegradable ingredients, low-toxicity, and nontoxic make them suitable as larvicides, repellents, insecticides, growth inhibitors and deterrents. (Chaudhari et al., 2021). The most harmful bug is the mosquito, which spreads illnesses including yellow fever, dengue, filariasis, malaria, and dengue fever and is responsible for millions of annual fatalities (Sofi et al., 2022). Controlling vectors carrying mosquito larvae is the only way to stop the spread of these illnesses. Often, this is achieved by consistently using larvicidal pesticides, particularly insect growth inhibitors and organophosphates (Rao et al., 2021). The frequent application of these chemical sprays has negative effects on populations that are not the intended target and may even cause resistance strain breakout (Zaller and Zaller, 2020). New, safer, and more efficient methods are required to control mosquito larvae. The first crystalline component isolated from karanja seed oil was karannjin (1.25%, oil basis), the main furanoflavonoid found in karanja seeds. A scalable procedure was developed in our lab to extract 95%–98% pure Karanjin from karanja cake that was removed (Satyvani et al., 2015; Singh et al., 2021). One of the vectors most often associated with both urban and rural human environments is the *C. quinquefasciatus* mosquito (Talipouo et al., 2021). In this study, we focused on the insect species *C. quinquefasciatus* and its distinctive C-terminus, which is shorter than that of *Bombyx mori* and Antheraea polyphemus. Our research revealed that CquiOBP1, a protein found in *C. quinquefasciatus*, and the oviposition pheromone (MOP) complex showed strong binding affinities at pH 7, but their interactions weakened at pH 5. Our findings suggest that mechanistic analysis of CquiOBP1 would be useful, as our theoretical and experimental results were in close agreement. Studies on botanical pesticides to identify natural alternatives to synthetic insecticides have recently been conducted. Several plants, including rice, mango (Dalavayi Haritha et al., 2021), and tomatoes, contain geranylacetone as an aromatic compound. Geranylacetone is produced when ozone degrades plant materials, along with other ketones (Al-Zharani et al., 2021).

Tyrosinase, a type III copper-containing enzyme, is important for producing the natural pigments of melanins. It is also referred to as monophenol or polyphenol oxidase (Ashooriha et al., 2020). There are structural differences in the enzymes among various organisms, which are conserved in melanin biosynthesis. Tyrosinase initiates melanin synthesis by oxidising tyrosine amino acids in two stages, resulting in the production of L-DOPA and dopaquinone (Cao et al., 2021). Tyrosinase is an essential enzyme that contributes to the process of enzymatic browning and regulates the metabolic pathway of melanin production. Melanin, a pigment generated during melanogenesis, is responsible for the colour of mammalian skin, eyes, and hair (Obaid et al., 2021). Tyrosinase catalyzes the hydroxylation of L-tyrosine converted to L-DOPA and L-DOPA converted to dopaquinones (Varghese et al., 2021). Dopaquinone produced within the body can be subjected to additional non-enzymatic polymerisation, resulting in the formation of eumelanin, which appears black, and pheomelanin, which has a brownish hue when reacted with thiol groups (Ito et al., 2020). Tyrosinase (EC 79 1.14.18.1), which plays a crucial role in initiating the biosynthetic melanin pathway, has been found in various species, including bacteria, fungi, plants, and animals (Epriliati, 2020). After isolating tyrosine 85 from *Rhizopus* nigricans, it was confirmed to be a precursor of melanin due to its unique properties. This was attributed to the presence of a different enzyme responsible for the oxidation of tyrosine, which was named “tyrosinase” by Bertrand, considering its unique characteristics (Yuan et al., 2020).

The chemical compound geranyl of acetone (GA) is also a natural perfume with notes of magnolia, 3,7-dimethyl-2,6-octadienyl acetone, and can be utilised in a variety of settings. It contributes up to 25.3% of the composition of Conyza bonariensis L. (20.3%) to the composition of the large yellow arrow and 13.7% to the composition of feldspar, as well as other essential oils. GA is employed in medicine as a synthetic vitamin and pharmaceutical intermediate because it exhibits substantial biological and antioxidant activities (Abbas et al., 2022). In mice low in food, Kawai observed that this had a recuperative effect on the mucosa of the stomach, which was damaged by heat shock (Walters, 2018).

The use of GA in the food sector and medicine is restricted by the fact that it is a viscous, volatile liquid that is difficult to dissolve in water, is white or yellowish-white in color, and is present at room temperature (Prasad et al., 2022). Expanding the use of GA requires an increase in its water solubility and stability, and their research investigated the effects of various adsorption materials on its adsorption properties (Du et al., 2020).

**FIGURE 1:**
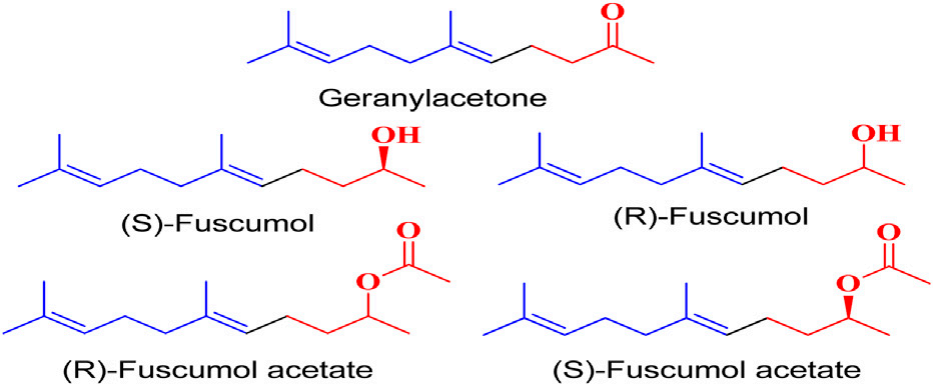
Terpeniod based compounds.

The discovery of terpenoid pheromones in species from North and South America belonging to the acanthocinini and acanthurini tribes, which are made up of certain enantiomers of related substances in nonracemic ratios, as well as the structurally related molecule certain 6-methylhept-5-en-2-one (sulcatone), nonracemic ratios of their enantiomers, 6,10-dimethyl-5,9- undecadien-2-ol (fuscumol), its acetate ester, 6-methylhept-5-en-2-ol may also be present in 6-methylhept-5-en-2-one (sulcatol), and 6,10-dimethyl-5,9- undecadien-2-yl acetate (fuscumol acetate) as shown in (Figure 1). Chemical pesticides are the primary methods used to decrease vector mosquito populations (Major et al., 2022), despite their limited availability due to environmental harm and non-target creatures (Mulkiewicz et al., 2021). The utilisation of chemical insecticides presents greater difficulties and a variety of possible environmental issues, including their widespread emergence and their resistance to and interference with organic biological regulation mechanisms (Reyes-Calderón et al., 2022). Geranyl acetone (GA) derivatives can help solve these issues by overcoming novel mosquito larval inhibitors and enhancing green techniques.

The environmental problems caused by these chemicals, the occurrence of mosquito species, and the disruption of natural biological control systems; require the selective control of mosquito larvae. Odourant-binding proteins transport odourants to olfactory receptors, which play a major role in host seeking. Based on the above information, we selected geranylacetone derivatives as targets against *C. quinquefasciatus* and used an odourant-binding protein (PDB ID: 3OGN) for molecular docking studies. In this study, we synthesized Mushroom Tyrosinase enzyme catalysis mediated to synthesis of new larvicidal active geranylacetone analogues Mannich base derivatives for evaluating their larvicidal activity.

## 2 Experimental

### 2.1 Materials and methods

All chemicals were purchased from Sigma-Aldrich. Nicolet iS5 (Thermo Scientific) was used for FTIR analysis (4,000–400 cm^−1^). ^1^H and ^13^C NMR spectra were recorded using Bruker DRX-300 and 75 MHz instruments, respectively. A Vario EL III elemental analyser was employed to determine the percentages (%) of assemblies (C, H, N, and S). The GC-MS Clarus SQ8 model (Perkin Elmer) was used to capture the mass spectra.

### 2.2 General procedure for the synthesis of compound 1a

The reaction mixture was substituted amine (0.01 mol), 3-methylcrotonaldehyde (0.01 mol, 0.70 mL), and geranylacetone (0.01 mol, 1.94 mL) with the tyrosinase enzyme were all mixed and grind in a mortar at ambient temperature at 30 min. The catalyst was then filtered to recover 2 mL of a 50 mM potassium phosphate buffer have been added. The residue was treated with water (30 mL), sodium bicarbonate (50 mL), and brine (30 mL), and washed successively. The solution was dried, Na_2_SO_4_ was added, and the solvent was evaporated. The final compound was identified by using thin-layer chromatography and then solid material was isolated and separated from column chromatography using silica gel and (ethyl acetate 4: hexane 6) to separate the solid material. The above procedure was used to synthesise other compounds (**1b–l**).

#### 2.2.1 4-(2-benzylidenehydrazinyl)-2,10,14-trimethylpentadeca-2,9,13-trien-6-one (1a)

White solid; mp: 119°C–114°C; IR (KBr) *ν* **:** 3172.39, 3061.59, 3039.24, 1712.01, 1621.15, 567.06cm^-1^; ^1^H NMR (300 MHz): *δ* 8.36 (s, -N=C, 1H), 7.83–7.52 (5H, -Ar ring, m), 5.20 (2H, t, *J* = 2.76), 5.33 (1H,-H, d, *J* = 3.21 Hz), 3.51 (1H, -CH, dd, *J* = 3.23Hz, *J* = 1.56 Hz), 2.84 (1H, -CH_2_, d, *J* = 3.45 Hz), 2.59 (1H, -CH_2_, d, *J* = 3.45 Hz), 2.46 (dd, -CH_2_, 2H, *J* = 2.32Hz, *J* = 3.45 Hz), 2.26 (dd, -CH_2_, 2H, *J* = 2.32Hz, *J* = 3.45 Hz), 1.94 (4H, -CH_2_, dd, *J* = 3.67Hz, *J* = 2.65 Hz), 1.82 (9H, -CH_3_, q, *J* = 4.23 Hz), 1.70 (6H, -CH_3_, d, *J* = 2.78 Hz), 2.12 (1H, -NH, s); ^13^C NMR (75 MHz): 210.8(-C=O), 143.3 (-C=N), 136.7 (-HC = CH-), 135.7 (-HC = CH-), 133.6, 131.7, 129.6, 128.9 (Ph), 132.0 (-HC = C-), 132.0, 128.5 (-HC = CH-), 124.3 (-HC = CH-), 123.5 (-HC = C-), 49.2, 45.7, 42.7, 39.7, 26.2, 24.3, 22.1, 21.0, 18.8, 16.2; EI-MS (*m/z*): 380.57 (M^+^, 27.5%); Anal. Calcd. for C_25_H_36_N_2_O: C, 78.90; H, 9.53; N, 7.36%; found: C, 78.93; H, 9.56; N, 7.34%.

#### 2.2.2 2,10,14-trimethyl-4-((*E*)-2-((*E*)-3-phenylallylidene)hydrazinyl)pentadeca-2,9,13-trien-6-one (1b)

Light green solid; mp: 141°C–144°C; IR (KBr) *ν* **:** 3172.67, 3071.57, 3030.44, 2592.56, 1713.16, 1623.44; ^1^H NMR (300 MHz): *δ* 7.45 (1H, -N=C, s), 7.65–7.29 (5H, -Ar ring, m), 5.18 (2H, t, *J* = 2.23), 5.30 (1H,-H, d, *J* = 3.21 Hz), 3.58 (1H, -CH, dd, *J* = 3.21Hz, *J* = 1.52 Hz), 2.82 (1H, -CH_2_, d, *J* = 3.42 Hz), 2.59 (1H, -CH_2_, d, *J* = 3.42 Hz), 2.47 (dd, 2H, -CH_2_, *J* = 2.32 Hz, *J* = 3.42 Hz), 2.24 (dd, 2H, -CH_2_, *J* = 2.32 Hz, *J* = 3.45 Hz), 1.92 (4H, -CH_2_, dd, *J* = 3.67Hz, *J* = 2.65 Hz), 1.81 (9H, -CH_3_, q, *J* = 4.23 Hz), 1.70 (6H, -CH_3_, d, *J* = 2.78 Hz), 7.32 (1H, -NH, s), 6.83 (1H, -CH), 7.22 (1H, -CH); ^13^C NMR (75 MHz): 210.2 (-C=O), 137.2(-C=N), 126.3 (-HC = CH-), 134.4 (-HC = CH-), 135.7, 128.4,128.3, 127.2 (Ph), 132.3 (-HC = C-), 128.1 (-HC = CH-), 124.2 (-HC = CH-), 123.2 (-HC = C-), 49.3 (-CH-), 45.2, 42.1, 39.5, 26.0, 24.5, 22.0, 21.2, 18.9, 16.2; EIMS(*m/z*) 406.60 (M^+^,30.4%); Anal. Calcd. for C_27_H_38_N_2_O: C, 79.76; H, 9.42; N, 6.89%; found: C, 79.73; H, 9.47; N, 6.88%.

#### 2.2.3 2,10,14-trimethyl-4-(2-phenylhydrazinyl)pentadeca-2,9,13-trien-6-one (1c)

Light green solid; mp: 147°C–151°C; IR (KBr) *ν:* 3174.44, 3071.58, 3034.27, 2592.38, 1711.02, 1621.38 cm^-1^; ^1^H NMR (300 MHz): *δ* 7.39–6.82 (5H, -Ar ring, m), 5.22 (2H, t, *J* = 2.76), 5.39 (1H,-H, d, *J* = 3.21 Hz), 3.52 (1H, -CH, dd, *J* = 3.23 Hz, *J* = 1.56 Hz), 2.86 (1H, -CH_2_, d, *J* = 3.45 Hz), 2.61 (1H, -CH_2_, d, *J* = 3.45 Hz), 2.56 (dd, 2H, -CH_2_, *J* = 2.33Hz, *J* = 3.41 Hz), 2.27 (dd, -CH_2_, 2H, *J* = 2.33Hz, *J* = 3.41 Hz), 1.94 (4H, -CH_2_, dd, *J* = 3.67Hz, *J* = 2.65 Hz), 1.80 (9H, -CH_3_, q, *J* = 4.23 Hz), 1.71 (6H, -CH_3_, d, *J* = 2.78 Hz), 4.58 (1H, NH), 2.14 (1H, -NH, s); ^13^C NMR (75 MHz): 211.7 (-C=O), 143.7 (-C=N), 138.9 (-HC = CH-), 135.8 (-HC = CH-), 124.1, 127.5, 115.1, 152.9 (Ph), 134.3 (-HC = C-), 129.1 (-HC = CH-), 123.7 (-HC = CH-), 124.5 (-HC = C-), 48.9 (-CH), 45.1, 42.1, 39.2, 26.2, 24.5, 22.7, 21.2, 18.9, 16.0; EIMS(*m/z*): 368.56 (M^+^, 26.4%); Anal. Calcd. for C_24_H_36_N_2_O: C, 78.21; H, 9.85; N, 7.60%; found: C, 78.24; H, 9.88; N, 7.57%.

#### 2.2.4 (*E*)-2,10,14-trimethyl-4-(phenylamino)pentadeca-2,9,13-trien-6-one (1d)

White Solid; mp: 147°C–149°C; IR (KBr) *ν* **:** 3172.57, 3071.44, 3034.19, 1713.21, and 1621.28 cm^−1^; ^1^H NMR (300 MHz): *δ* 8.36(-N=C, 1H, s), 7.34–6.75 (5H, -Ar ring, m), 5.33 (1H,-H, d, *J* = 3.23 Hz), 5.21 (2H, t, *J* = 2.76), 4.62 (1H, -NH, s), 3.67 (1H, -CH, dd, *J* = 3.23Hz, *J* = 1.56 Hz), 2.84 (1H, -CH_2_, d, *J* = 3.45 Hz), 2.59 (1H, -CH_2_, d, *J* = 3.45 Hz), 2.47 (-CH_2_, 2H, dd, *J* = 2.32Hz, *J* = 3.45 Hz), 2.22 (-CH_2_, 2H, dd, *J* = 2.32Hz, *J* = 3.45 Hz), 1.92 (4H, -CH_2_, dd, *J* = 3.67Hz, *J* = 2.65 Hz), 1.83 (8H, -CH_3_, q, *J* = 4.23 Hz), 1.74 (6H, -CH_3_, d, *J* = 2.78 Hz); ^13^C NMR (75 MHz): 209.1 (-C=O), 143.7 (-C=N), 136.9 (-HC = CH-), 134.2 (-HC = CH-), 147.8, 117.3, 127.1, 197.0 (Ph), 133.9 (-HC = C-), 128.2 (-HC = CH-), 124.0 (-HC = CH-), 123.4 (-HC = C-), 50.1 (-CH-), 45.9, 41.9, 38.2, 27.0, 23.9, 22.5, 21.2, 18.8, 16.0; EIMS(*m/z*): 353.54 (M^+^, 26.4%); Anal. Calcd. for C_24_H_35_NO: C, 81.53; H, 9.98; N, 3.96%; found: C, 81.54; H, 9.96; N, 3.94%.

#### 2.2.5 (*E*)-2,10,14-trimethyl-4-(p-tolylamino)pentadeca-2,9,13-trien-6-one (1e)

White solid; mp: 146°C–149°C; IR (KBr) *ν* **:** 3174.60, 3077.47, 3034.31, 1718.20, 1621.40 cm^−1^; ^1^H NMR (300 MHz): *δ* 7.09–6.58 (4H, -Ph-, dd), 5.36(1H,-H, d, *J* = 3.23 Hz), 5.24 (2H, s, -H), 4.39 (1H, -NH, s) 3.51 (1H, -CH, dd, *J* = 3.23 Hz, *J* = 1.56 Hz), 2.81 (1H, -CH_2_, d, *J* = 3.45 Hz), 22.50 (-CH_2_, 2H, dd, *J* = 2.32Hz, *J* = 3.40 Hz), 2.08 (-CH_2_, 2H, dd, *J* = 2.32Hz, *J* = 3.45 Hz), 2.00 (4H, -CH_2_, d, *J* = 3.45 Hz), 1.91 (4H, -CH_2_, dd, *J* = 3.67Hz, *J* = 2.65 Hz), 1.80 (9H, -CH_3_, q, *J* = 4.23 Hz), 1.72 (6H, -CH_3_, d, *J* = 2.78 Hz); ^13^C NMR (75 MHz): 211.3 (-C=O), 143.0 (-C=N), 135.3 (-HC = CH-), 134.2 (-HC = CH-), 146.5, 113.2, 129.0, 127.2 (Ph-CH_3_), 24.3 (-CH_3_), 133.9 (-HC = C-), 132.0, 128.5 (-HC = CH-), 124.0 (-HC = CH-), 123.5 (-HC = C-), 50.6 (-CH-), 45.1, 42.7, 39.5, 26.0, 24.7, 22.0, 21.7, 18.2, 15.9; EIMS(*m/z*): 367.57 (M^+^, 27.5%); Anal. Calcd. for C_25_H_37_NO: C, 81.69; H, 10.15; N, 3.81%; found: C, 81.65; H, 10.19; N, 3.86%.

#### 2.2.6 *N*-(2,10,14-trimethyl-6-oxopentadeca-2,9,13-trien-4-yl)benzamide (1f)

Yellow solid; mp: 145°C–139°C; IR (KBr) *ν* **:** 3179.10, 3070.42, 3033.30, 1711.07, and 1627.20; ^1^H NMR (300 MHz): *δ* 8.37(1H, -NH-CO-, s), 8.16–7.71 (5H, -Ar ring, m), 5.32 (1H,-H, d, *J* = 3.23 Hz), 5.20(2H, t, *J* = 2.76), 4.50 (dd, 1H, -CH, *J* = 3.23 Hz, *J* = 1.56 Hz), 2.81 (1H, -CH_2_, d, *J* = 3.45 Hz), 2.46 (-CH_2_, 2H, dd, *J* = 2.32 Hz, *J* = 3.45 Hz), 2.19 (2H, -CH_2_, dd, *J* = 2.32 Hz, *J* = 3.45 Hz), 2.14 (1H, -NH, s), 2.00 (4H, -CH_2_, d, *J* = 3.45 Hz), 1.87 (9H, -CH_3_, q, *J* = 4.23 Hz), 1.72 (6H, -CH_3_, d, *J* = 2.78 Hz); ^13^C NMR (75 MHz): 168.6 (-NH-CO-), 211.3 (-C=O), 144.1 (-C=N), 135.2 (-HC = CH-), 135.2 (-HC = CH-), 134.5, 131.7, 128.9, 127.4 (Ph), 132.1 (-HC = C-), 132.3, 128.5 (-HC = CH-), 124.2, 123.6, 42.8 (-CH-), 45.1, 42.2, 39.3, 26.2, 24.3, 22.6, 21.2, 18.9, 16.2; EIMS(*m/z*): 381.27 (M^+^, 27.5%); Anal. Calcd. for C_25_H_35_NO_2_: C, 78.70; H, 9.25; N, 3.67%; found: C, 78.73; H, 9.21; N, 3.62%.

#### 2.2.7 (*E*)-4-((Z)-2-(furan-2-ylmethylene)hydrazinyl)-2,10,14-trimethylpentadeca-2,9,13-trien-6-one (1g)

Yellow solid; mp: 153°C–158°C; IR (KBr) *ν* **:** 3172.41, 3070.31, 3032.27, 1719.11, 1621.31; ^1^H NMR (300 MHz): *δ* 8.36 (1H, -N=C, s), 7.71 (1H, -Fural ring, d), 6.92 (1H, -Fural ring, d), 6.49 (1H, -Fural ring, dd), 5.34 (1H, -H, d, *J* = 3.23 Hz), 5.29 (2H, t, *J* = 2.76), 3.51 (1H, -CH, dd, *J* = 3.23 Hz, *J* = 1.56 Hz), 2.84 (-CH_2_, 1H, d, *J* = 3.45 Hz), 2.59 (-CH_2_, 1H, d, *J* = 3.45 Hz), 2.47 (2H, -CH_2_, dd, *J* = 2.32 Hz, *J* = 3.45 Hz), 2.20 (-CH_2_, 2H, dd, *J* = 2.32 Hz, *J* = 3.45 Hz), 1.92 (4H, -CH_2_, dd, *J* = 3.67 Hz, *J* = 2.65 Hz), 1.83 (9H, -CH_3_, q, *J* = 4.23 Hz), 1.71 (6H, -CH_3_, d, *J* = 2.78 Hz), 7.48 (1H, -NH, s); ^13^C NMR (75 MHz): 147.3, 118.1, 112.3, 143.1, 210.1 (-C=O), 136.5 (-C=N), 136.7 (-HC = CH-), 135.7 (-HC = CH-), 133.5, 131.7, 129.6, 128.9(Ph), 132.0 (-HC = C-), 132.0, 128.2 (-HC = CH-), 124.3 (-HC = CH-), 123.5 (-HC = C-), 49.1, 45.5, 42.7, 39.7, 26.2, 24.3, 22.1, 21.0, 18.8, 16.2; EIMS(*m/z*): 370.53 (M^+^, 25.3%); Anal. Calcd. for C_23_H_34_N_2_O_2_: C, 74.55; H, 9.25; N, 7.56%; found: C, 74.53; H, 9.27; N, 7.52%.

#### 2.2.8 (E)-4-((Z)-2-(4-(dimethylamino)benzylidene)hydrazinyl)-2,10,14-trimethylpentadeca-2,9,13-trien-6-one (1 h)

Yellow solid; mp: 149°C–151°C; IR (KBr) *ν* **:** 3171.20, 3073.38, 3032.31, 1715.17, 1624.40; ^1^H NMR (300 MHz): *δ* 8.34 (1H, -N=C, s), 7.80–7.53 (5H, -Ar ring, m), 5.22 (2H, t, *J* = 2.76), 5.36 (2H, -H, d, *J* = 3.23 Hz), 3.51 (2H, -CH, dd, *J* = 3.21 Hz, *J* = 1.56 Hz), 2.84–2.59 (2H, -CH_2_, d, *J* = 3.45 Hz), 2.43 (-CH_2_, 2H, dd, *J* = 2.32 Hz, *J* = 3.45 Hz), 2.22 (-CH_2_, 2H, dd, *J* = 2.32 Hz, *J* = 3.45 Hz), 2.00 (4H, -CH_2_, d, *J* = 3.45 Hz), 1.93 (4H, -CH_2_, dd, *J* = 3.67Hz, *J* = 2.65 Hz), 1.85 (9H, -CH_3_, q, *J* = 4.23 Hz), 1.76 (6H, -CH_3_, d, *J* = 2.78 Hz); ^13^C NMR (75 MHz): 210.8 (-C=O), 144.8 (-C=N), 137.6 (-HC = CH-), 134.3 (-HC = CH-), 133.0, 131.2, 128.2, 127.2 (Ph), 132.9 (-HC = C-), 127.4 (-HC = CH-), 123.8 (-HC = CH-), 122.2 (-HC = C-), 48.6, 43.9, 42.7, 37.9, 25.2, 24.5, 22.7, 21.3, 18.5, 16.2; EIMS(*m/z*): 424.63 (M^+^, 29.7%); Anal. Calcd. for C_27_H_41_N_3_O: C, 76.55; H, 9.76; N, 9.92%; found: C, 76.57; H, 9.81; N, 9.94%.

#### 2.2.9 (E)-4-((Z)-2-(4-chlorobenzylidene)hydrazinyl)-2,10,14-trimethylpentadeca-2,9,13-trien-6-one (1i)

Yellow solid; mp: 165°C–169°C; IR (KBr) *ν* **:** 3171.18, 3073.24, 3032.39, 1715.10, 1624.47; ^1^H NMR (300 MHz): *δ* 8.38 (1H, -N=C, s), 7.72–7.49 (4H, -Ar ring, dd), 5.20 (2H, t, *J* = 2.76), 5.33 (1H,-H, d, *J* = 3.21 Hz), 3.51 (1H, -CH, *J* = 3.23Hz, *J* = 1.56 Hz, dd), 2.84 (1H, -CH_2_, d, *J* = 3.45 Hz), 2.59 (1H, -CH_2_, d, *J* = 3.45 Hz), 2.43 (-CH_2_, 2H, dd, *J* = 2.32Hz, *J* = 3.45 Hz), 2.24 (-CH_2_, 2H, dd, *J* = 2.32Hz, *J* = 3.45 Hz), 2.13 (1H, -NH, s), 1.92 (4H, -CH_2_, dd, *J* = 3.67 Hz, *J* = 2.65 Hz), 1.80 (9H, -CH_3_, q, *J* = 4.23 Hz), 1.69 (6H, -CH_3_, d, *J* = 2.78 Hz); ^13^C NMR (75 MHz): 212.1 (-C=O), 143.8 (-C=N), 135.2 (-HC = CH-), 134.0 (-HC = CH-), 136.4, 128.6, 139.5, 132.6 (Ph-Cl), 132.0 (-HC = C-), 128.2 (-HC = CH-), 123.1 (-HC = CH-), 122.5 (-HC = C-), 49.8, 45.1, 43.0, 38.2, 27.1, 24.3, 22.0, 21.2, 18.3, 16.4; EIMS(*m/z*): 416.01 (M^+^, 32.2%); Anal. Calcd. for C_25_H_35_ClN_2_O: C, 72.35; H, 8.50; Cl, 8.54; N, 6.75%; found: C, 72.31; H, 8.52; Cl, 8.49; N, 6.72%.

#### 2.2.10 (E)-2,10,14-trimethyl-4-((Z)-2-(3-methylbut-2-en-1-ylidene)hydrazinyl)pentadeca-2,9,13-trien-6-one (1j)

Yellow solid; mp: 123°C–127°C; IR (KBr) *ν* **:** 3171.24, 3071.45, 3032.20, 1715.19, 1624.39; ^1^H NMR (300 MHz): *δ* 7.58 (1H, -N=C, s), 7.12 (1H, -NH, s), 5.37 (1H,-H, d, *J* = 3.23 Hz), 5.26 (2H, t, *J* = 2.76), 4.80 (-CH, 1H, d), 3.51 (-CH, 1H, dd, *J* = 3.23Hz, *J* = 1.56 Hz), 2.59 (1H, -CH_2_, d, *J* = 3.47 Hz), 2.84 (1H, -CH_2_, d, *J* = 3.47 Hz), 2.41 (-CH_2_, 2H, dd, *J* = 2.32 Hz, *J* = 3.45 Hz), 2.29 (-CH_2_, 2H, dd, *J* = 2.32 Hz, *J* = 3.47 Hz), 2.13 (3H, -CH_3_, s), 1.94 (4H, -CH_2_, dd, *J* = 3.67Hz, *J* = 2.65 Hz), 1.91 (3H, -CH_3_, s), 1.82 (9H, -CH_3_, q, *J* = 4.23 Hz), 1.72 (6H, -CH_3_, d, *J* = 2.78 Hz); ^13^C NMR (75 MHz): 211.2 (-C=O), 152.3 (-C(CH_3_)_2_), 26.7 ((-CH_3_)_2_), 138.1 (-HC = N-), 136.4 (-HC = CH-), 135.1 (-HC = CH-), 123.4 (-CH), 132.0 (-HC = C-), 127.2 (-HC = CH-), 125.2 (-HC = CH-), 123.1 (-HC = C-), 47.2 (-CH-), 45.1, 42.3, 39.6, 26.3, 23.2, 22.0, 21.2, 18.2, 16.3; EIMS(*m/z*): 358.56 (M^+^, 26.1%); Anal. Calcd. for C_23_H_38_N_2_O: C, 77.04; H, 10.68; N, 7.81%; found: C, 77.13; H, 10.61; N, 7.84%.

#### 2.2.11 (E)-2,10,14-trimethyl-4-((Z)-2-(3-methylbut-2-en-1-ylidene)hydrazinyl)pentadeca-2,9,13-trien-6-one (1 k)

Yellow solid; mp: 123°C–127°C; IR (KBr) *ν* **:** 3171.21, 3075.45, 3032.23, 1715.19, 1624.39; ^1^H NMR (300 MHz): *δ* 7.0 (1H, s, -CH), 7.50 (1H, s, -CH)5.33 (1H, d, *J* = 3.23 Hz), 5.22 (2H, t, *J* = 2.76), 4.84 (1H, -CH, d), 3.51 (1H, -CH, dd, *J* = 3.23 Hz, *J* = 1.56 Hz), 2.84 (2H, -CH_2_, d, *J* = 3.45 Hz), 2.47 (-CH_2_, 2H, dd, *J* = 2.32 Hz, *J* = 3.45 Hz), 2.25 (-CH_2_, 2H, dd, *J* = 2.32 Hz, *J* = 3.45 Hz), 2.16 (3H, -CH_3_, s), 2.00 (4H, -CH_2_, d, *J* = 3.45 Hz), 1.92 (3H, -CH_3_, s), 1.81 (9H, -CH_3_, q, *J* = 4.23 Hz), 1.72 (6H, -CH_3_, d, *J* = 2.78 Hz); ^13^C NMR (75 MHz): 211.1 (-C=O), 136.9 (-HC = N-), 136.1 (-HC = CH-), 135.2 (-HC = CH-), 132.2, 123.2, 128.1, 123.0, 121.0 (-HC = CH-), 121.2 (-HC = C-), 50.6 (-CH-), 47.2, 42.3, 39.3, 32.1 (-CH_3_), 26.1, 24.2, 22.1, 21.0, 18.6, 16.6; EIMS(*m/z*): 358.56 (M^+^, 26.1%); Anal. Calcd. for C_23_H_38_N_2_O: C, 77.04; H, 10.68; N, 7.81%; found: C, 77.06; H, 10.67; N, 7.83%.

#### 2.2.12 (E)-4-((4-bromophenyl)amino)-2,10,14-trimethylpentadeca-2,9,13-trien-6-one (1l)

Yellow solid; mp: 138°C–141°C; IR (KBr) *ν* **:** 3171.17, 3071.33, 3032.24, 1715.13, 1624.32; ^1^H NMR (300 MHz): *δ* 7.13–6.59 (4H, -Ph ring, dd), 5.21 (2H, t, *J* = 2.76 Hz), 5.35 (1H,-H, d, *J* = 3.27 Hz), 3.51 (1H, -CH, dd, *J* = 3.23Hz, *J* = 1.56 Hz), 2.84 (1H, -CH_2_, d, *J* = 3.45 Hz), 2.59 (1H, -CH_2_, d, *J* = 3.45 Hz), 2.43 (-CH_2_, 2H, dd, *J* = 2.32 Hz, *J* = 3.45 Hz), 2.22 (-CH_2_, 2H, dd, *J* = 2.32 Hz, *J* = 3.45 Hz), 1.98 (4H, -CH_2_, dd, *J* = 3.67 Hz, *J* = 2.65 Hz), 1.84 (9H, -CH_3_, q, *J* = 4.23 Hz), 1.73 (6H, -CH_3_, d, *J* = 2.78 Hz), 4.32 (1H, -NH, s); ^13^C NMR (75 MHz): 210.8 (-C=O), 136.7 (-HC = CH-), 135.7 (-HC = CH-), 132.7, 114.8, 115.0, 145.7 (Ph), 133.6 (-HC = C-), 131.0, 127.1 (-HC = CH-), 124.9 (-HC = CH-), 122.2(-HC = C-), 50.4 (-CH-), 45.1, 42.2, 39.4, 25.1, 23.0, 22.4, 21.6, 18.1, 16.0; EIMS(*m/z*): 433.18 (M^+^, 97.4%); Anal. Calcd. for C_24_H_34_BrNO: C,66.66; H, 7.92; Br, 8.48; N, 3.24%; found: C,66.69; H, 7.94; Br, 8.43; N, 3.27%.

## 3 Biological screening

### 3.1 Larvicidal activity

The larvicidal activity was carried out using a previously described procedure. The urban mosquito larvae (*C. quinquefasciatus*) were tested against the synthesised compounds using a standard bioassay procedure. *Culex quinquefasciatus* eggs were obtained from a local drainage system. The larval hatching was accomplished by immersing the eggs in clean water at a temperature of the room. Larval growth was monitored for up to 7 days, and second instar larvae were collected and placed in a test vial. To determine larval mortality, various concentrations (25–100 μg/mL) of compounds (**1a-l**) were prepared. We evaluated larval mortality by determining the ratios (as a percentage) of dead and living larvae resulting from exposure to the compounds. To calculate the LD_50_ values, we used probit scale analysis (Sathishkumar et al., 2020; Al-Zharani et al., 2022). After 24 h, the number of surviving larvae was recorded, and test was carried out three times, that ensure the statistical reliability of the results. The mortality percentage of the synthesised compounds (**1a-l**) at the selected concentrations was estimated using a standard bioassay procedure (World Health Organization, 1981).

### 3.2 Molecular docking analysis

#### 3.2.1 Ligand preparation

Chemdraw 12.0, and Chem3Dpro software were used for the preparation of ligand molecules.

#### 3.2.2 Receptor preparation

Crystal structure of an odourant-binding protein from the Southern House Mosquito Complexed with an Oviposition Pheromone. The structure obtained from the protein data bank (PDB ID: 3OGN) PDB DOI: https://doi.org/10.2210/pdb3OGN/pdb was used in this study. Discovery Studio 2019 was employed to remove water molecules and ligands from the receptor. The SWISS PDB Viewer software was utilized to minimize the energy consumption of the receptor during the molecular docking analysis.

#### 3.2.3 The localization of the binding pocket

The Discovery Studio 2019 program was used to study the binding pocket of the target protein and co-crystallised ligand. Residues Phe123 was present in the binding pocket.

#### 3.2.4 Docking

AutoDock Vina 1.1.2 was used to analyse the molecular docking studies among the most effective geranyl acetone derivatives series, compound **1b**, permethrin compounds, and protein 3OGN. The active amino acid residues in the binding pocket formed a docking grid box. The Pymol and Discovery studio 2019 programming were used to visually analyse the interactions with the exhaustiveness value set to 8 (Mullaivendhan et al., 2023; Loganathan et al., 2024).

### 3.3 Larval growth inhibition and regulation

The compound **1b** (10 μg/mL) was tested for larval growth in *C. Quinquefasciatus* and evaluated using the water immersion method, the method was followed by previously reported our studies (Chidambaram et al., 2021).

### 3.4 Antifeedant activity - ichthyotoxicity for non -aquatic target for the toxicity measurement

All compounds (**1a-l**) were tested for antifeedant activity evaluated for marine fngerlings (*Oreochromis mossambicus*). The test compounds were used for various concentrations 10, 25, 50 and 100 μg/mL. Mortality was analysis the ratios (%) of the numbers of dead and live fingerlings in screening, the method was followed by described previously (Sathishkumar et al., 2020; Chidambaram et al., 2021). The toxicity values are summarized in (Table 4).

### 3.5 Statistical analysis

The LD_50_ value was determined by aggregating the outcomes of a minimum of three independent experiments, and the standard deviations (SD) were determined using Microsoft Excel.

## 4 Results and discussion

### 4.1 Chemistry

The grindstone method has been found to be more efficient and selective in certain cases compared to traditional organic reactions (Abdel Hameed, 2015). Furthermore, this method has several benefits including low cost, rapid reaction time, high reproducibility, simplicity of process, minimal pollution, and mild reaction conditions (Li et al., 2011). Grindstone chemistry was received growing interest in recent years owing to its potential for greener organic transformations. In this study, we used the grinding stone method to synthesize target compounds by mixing the reaction mixture of substituted amine (0.01 mol), 3-methylcrotonaldehyde (0.01 mol, 0.70 mL), and geranylacetone (0.01 mol, 1.94 mL) with the tyrosinase enzyme in a mortar at ambient temperature. The mixture was ground for 30 min at room temperature. The solvent was evaporating, and then solid material was isolated and separated from column chromatography. The final product was purified with suitable alcohol. The resulting solid material was then extracted using column chromatography with 4:6 ethyl acetate hexane.

**SCHEME 1:**
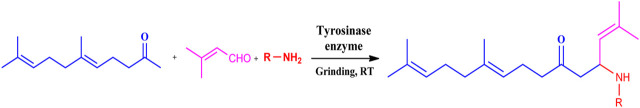
Synthetic method for producing **(1a–l)** geranylacetone analogues.

**SCHEME 2:**
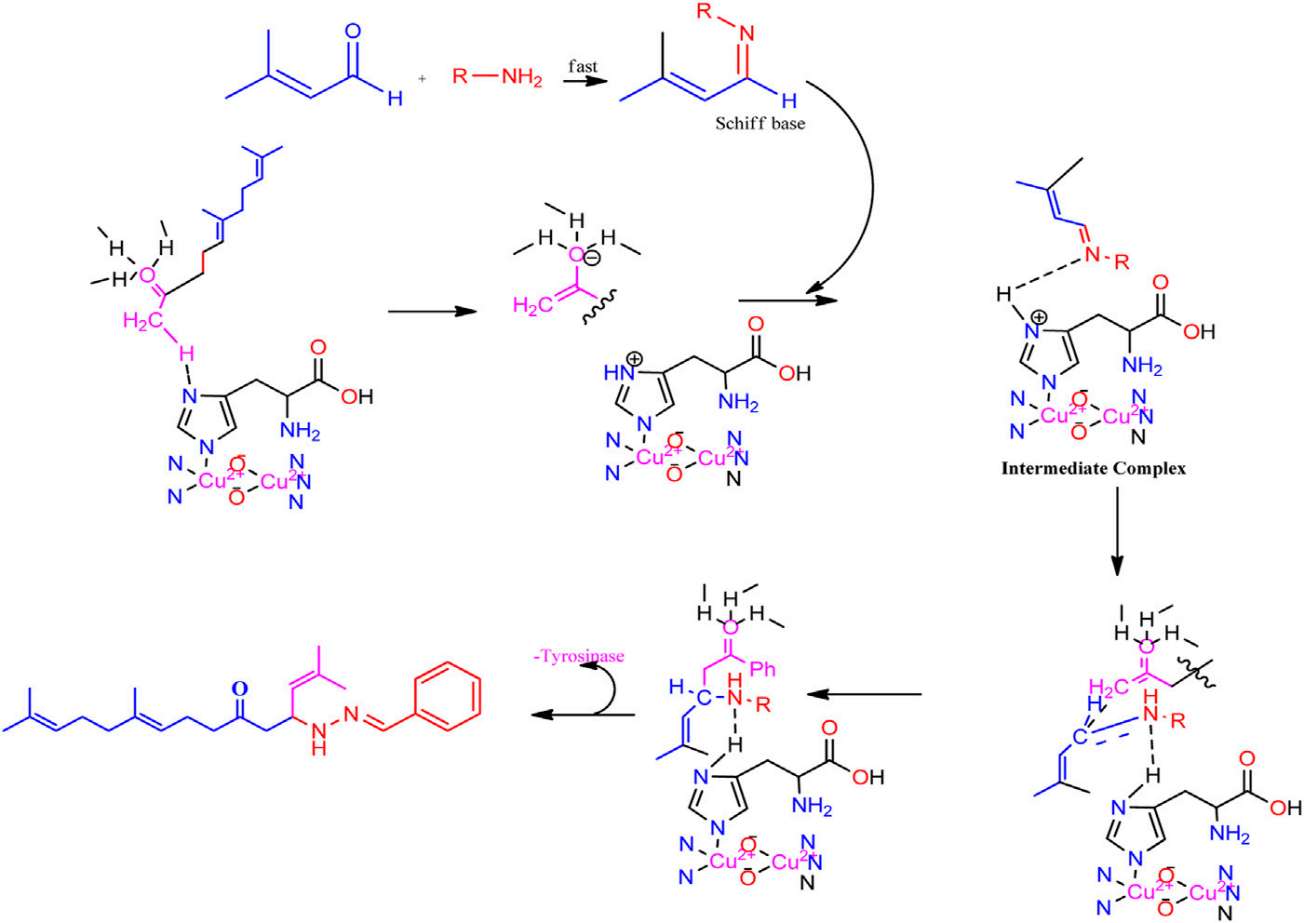
Speculated process for the synthesis of geranylacetone.

Geranylacetone analogues were produced using the grindstone method. Geranylacetone, 3-methylbut-2-enal, substituted amine, and the tyrosinase enzyme Cu(II) were mixed in a mortar and pestle. Subsequently, column chromatography was performed for purification. The general design of this synthetic pathway is illustrated in (Scheme 1). A representation of the chemical optimizations is displayed in (Table 1). The Cu(II)-tyrosinase-catalysed Mannich reaction has a proposed mechanism (Scheme 2). Initially, when combined, amine and aldehyde quickly combine for Schiff base generation, and at the same time, the tyrosinase enzyme pre-activates the ketone to create the enolate anion. Second, the basis for Schiff could create an intermediary with the complex of the tyrosinase residue. Materials with copper content, such as copper triflate (Chan et al., 2017), copper acetate (Jin et al., 2022), copper bromide (Javaid et al., 2022), and copper nanoparticles (Ouygang et al., 2022), are essential for Mannich base reactions. Many enzymes, including trypsin (Momeni et al., 2022), lipase (Shomal et al., 2022), and proteases (Heng et al., 2022), catalyse the one-pot multicomponent Mannich process. In the current study, the synthesis of a Mannich base (**1a–l**) derivative was catalysed by copper, which contains the Cu(II)-tyrosinase enzyme.

**TABLE 1:** Optimization of reactants and yield with final product for compounds (**1a-l)**.

Com.No.	NH_2_-R	Final products	Yield (%)
**1a**	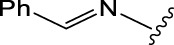	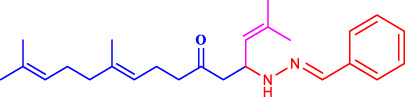	91
**1b**	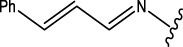	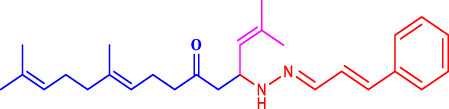	83
**1c**	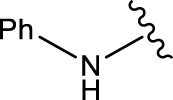	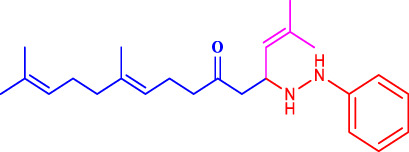	87
**1d**	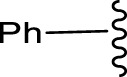	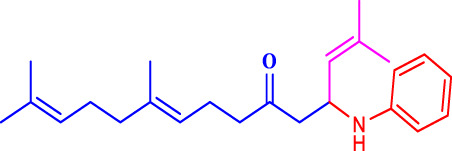	91
**1e**	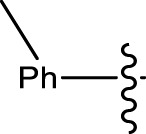	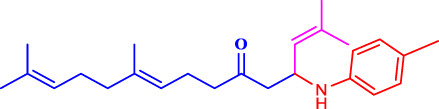	84
**1f**	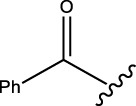	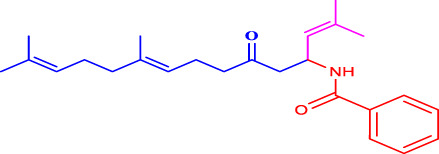	83
**1g**	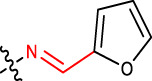	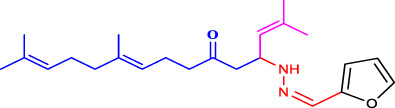	75
**1h**	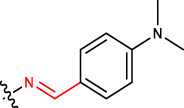	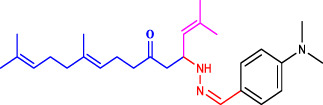	81
**1i**	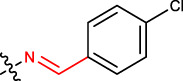	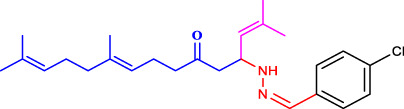	78
**1j**	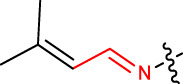	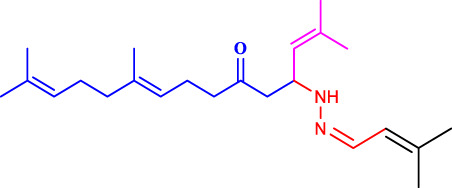	80
**1k**	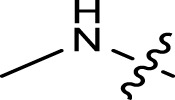	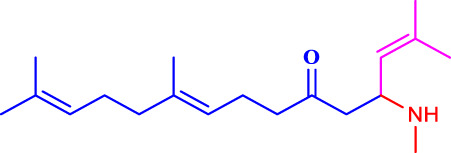	76
**1l**	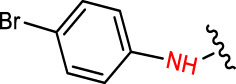	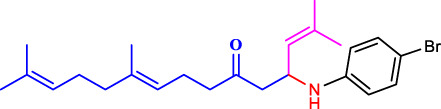	81

The compounds identified important bands at 3170.23 in the IR spectrum, 3176.54, 2595.45–2599.98, and 1710.68–1716.70 cm^-1^ in accordance with the -C=O, -NH, and -C=N groups. ^1^H, ^13^C NMR and FT-IR spectroscopy were used to identify the newly synthesized compounds. ^1^H NMR of the protons -NH, -CH, -CH_2_, -CH_3,_ and -Ph ring signal ranged from *δ* 7.12–2.12, 4.82–.51, 2.84–1.98, 1.87–1.72, and 7.83–6.75 ppm. The peaks in the ^13^C NMR spectrum between 132.0–121.2, 136–123.0, 212.1–209.1 and 143.3–136.5 ppm, which are associated with the = HC-C-, =HC-CH-, -C=O and–C=N- atoms respectively, were found. The conformation of each compound was identified using mass spectra and elemental analyses. With only a slight loss in the catalytic activity, at least ten runs were completed using the recovered catalyst. As regeneration or reaction occurs, there may be a slight loss of essential sites or a reduction in the catalytic surfaces. The recyclability of the enzyme catalyst, Cu(II)-tyrosinase, is shown in (Table 2). The molecular weight of all compounds was confirmed by EI-MS spectral analysis. The molecular ion **(**m/z) of **1a**: 380.18 (M^+^, 16.8%) was observed and conformed the molecular weight of the compound **1a**.

**TABLE 2:** Recyclability of the enzyme catalyst Cu(II)-tyrosinase.

Entry	Catalyst	Yield (%)
1	1st use	92
2	1st use	92
3	1st use	90
4	1st use	90
5	1st use	88
6	1st use	87
7	1st use	87
8	1st use	86
9	1st use	86
10	1st use	85

### 4.2 Biological activities

#### 4.2.1 Larvicidal activity

The geranylacetone derivatives (**1a–l**) were tested for activity against second-instar larvae of *C. quinquefasciatus*. Compounds **1c, 1d, 1f,** and **1l** had very low activity (LD_50_ > 100 μg/mL), even at 100 μg/mL. The compound **1a (**LD_50_: 59.4 μg/mL) was 80.2% active at 100 μg/mL, whereas the compound **1b** was highly active (LD_50_: 20.7 μg/mL) and 100% mortality reached at 100 μg/mL. Compound **1e** (LD_50_: 66.0 μg/mL) showed 60.4% activity at 100 μg/mL. For compound **1g** (LD_50_: 33.7 μg/mL) was 100% of active at 100 μg/mL, and compound **1h** (LD_50_: 46.7 μg/mL) was 100% activity reached at 100 μg/mL.

Compound **1i** displayed 48.6% activity with (LD_50_: 97.4 μg/mL) and the compound **1j** (LD_50_: 71.8 μg/mL) was 59.6% reached at 100 μg/mL. Compound 1 k displayed 63.2% activity at 100 μg/mL with an (LD_50_: 66.0 μg/mL). Among the synthesised compounds, compound **1b** showed higher activity than the other chemicals, outperforming permethrin. Compound **1g** was also highly active, but less active than compound **1b.** Compound **1b** was mutagenic and caused a death rate due to the presence of long chin unsaturated compounds, whereas the furan ring presence in compound **1g** was also highly responsive in this system. Given that they showed no active or harmful behaviour, it is possible that the presence of the geranylacetone analogue groups is responsible for the observed biological effect. Based on the aforementioned investigation, compound **1b** was found to be significantly active during larvicidal screening. The rate percentages and LD50 values are shown in Table 3.

**TABLE 3:** Larvicidal activity of compounds **(1a-l)**.

Compounds	% of mortality			LD_50_ (µg/mL)^a^
	25 μg/mL	50 μg/mL	100 μg/mL	
**1a**	24.1 ± 0.2	43.2 ± 0.1	80.2 ± 0.2	59.4
**1b**	52.0 ± 0.1	77.3 ± 0.8	100 ± 0.0	20.7
**1c**	19.3 ± 0.4	26.3 ± 0.4	40.2 ± 0.6	>100
**1d**	0 ± 0.0	0 ± 0.0	0 ± 0.0	>100
**1e**	33.3 ± 0.1	48.3 ± 0.2	60.4 ± 0.2	66.0
**1f**	49.2 ± 0.2	53.1 ± 0.2	40.1 ± 0.1	>100
**1g**	45.3 ± 0.2	59.3 ± 0.2	100 ± 0.0	33.7
**1h**	36.2 ± 0.1	48.3 ± 0.1	92.2 ± 0.1	46.7
**1i**	12.3 ± 0.1	36.2 ± 0.1	48.6 ± 0.3	97.4
**1j**	26.3 ± 0.2	47.3 ± 0.2	59.6 ± 0.2	71.8
**1k**	32.1 ± 0.2	45.3 ± 0.2	63.2 ± 0.1	66.0
**1l**	12.3 ± 0.2	26.3 ± 0.2	36.3 ± 0.3	>100
**Geranylacetone**	10.8 ± 0.6	22.8 ± 0.6	42.3 ± 0.6	>100
**Permethrin**	51.1 ± 1.0	76.3 ± 0.1	100 ± 0.0	24.4

#### 4.2.2 Regulation of larval growth

To investigate the effect of compound **1b** formulations on the growth, metamorphosis, and production of *C. quinquefasciatus* larvae for 72 h (Table 4). summarises the compound **1b** larval weight and growth inhibition effects. The eclosion rate, pupal time, and adult period of *C. quinquefasciatus* treated with compound **1b** (10 μg/mL) are shown in (Table 5). Compound **1b** showed 38.81% growth inhibition and controlled larval development. In addition, compound **1b** had a minimal impact of 52.94% eclosion rate in both the adult and pupal periods. Based on these results, compound **1b** was found to inhibit the growth of the *C. quinquefasciatus* larvae.

**TABLE 4:** Compound **1b** on the growth of *Culex quinquefasciatus*.

Compound	Weight			Weight gain (mg)	Inhibition (%)
	0 h		72 h		
1b^a^	100.6 ± 0.8		105.1 ± 0.2	4.5 ± 0.3	38.81 ± 0.27
Control^b^	100.5 ± 0.8		107.1 ± 0.2	6.6 ± 0.3	-

^a^ The concentration of 1b was 10 μg/mL.

^b^ Control is not containing the compounds.

**TABLE 5:** Compound **1b** on the development and growth of *Culex quinquefasciatus*.

Compound	Duration of pupae (h)	Duration of adult (h)	Rate of eclosion (%)
1b^a^	68.1 ± 0.24	23.1 ± 1.16	52.94 ± 1.06
Control^b^	61.34 ± 1.23	25.23 ± 1.06	80.67 ± 1.12

^a^ The concentration of 1b was 10 μg/mL.

^b^ Control is not containing the compounds.

#### 4.2.3 Antifeedant activity

All compounds (**1a-1l**) were effective against *C. quinquefasciatus*, whereas all compounds were less active against the antifeedant (marine fish Oreochromis), which is a non-aquatic target for toxicity measurement that indicates 0% mortality for most compounds, and all compounds reached the LD50 value at >100 μg/mL, which indicated no toxicity in water.

Compounds **1b, 1d,** and **1k** showed 0% mortality at 100 μg/mL. Geranylacetone derivatives were observed to have biological effects with no active toxic behaviour. The effects of these compounds on mortality are shown in (Table 6).

**TABLE 6:** Antifeedant activity of compounds **(1a–1l),** non -aquatic target for the toxicity measurement against marine fish Oreochromis.

Compounds	%Of mortality at 25 μg/mL	%Of mortality at 50 μg/mL	%Of mortality at 100 μg/mL	LD_50_ (µg/mL)^a^
**1a**	0.0 ± 0.0	2.0 ± 0.1	8.2 ± 0.1	>100
**1b**	0.0 ± 0.0	0.0 ± 0.0	0.0 ± 0.0	>100
**1c**	0.0 ± 0.0	0.0 ± 0.0	2.2 ± 0.6	>100
**1d**	0.0 ± 0.0	0.0 ± 0.0	0.0 ± 0.0	>100
**1e**	0.0 ± 0.0	7.3 ± 0.2	12.6 ± 0.4	>100
**1f**	0.0 ± 0.0	0.0 ± 0.0	4.1 ± 0.3	>100
**1g**	0.0 ± 0.0	0.0 ± 0.0	1.3 ± 0.2	>100
**1h**	2.4 ± 0.3	8.3 ± 0.1	16.2 ± 0.1	>100
**1i**	0.0 ± 0.0	6.2 ± 0.3	14.6 ± 0.4	>100
**1j**	3.9 ± 0.3	9.4 ± 0.4	23.6 ± 0.1	>100
**1k**	0.0 ± 0.0	0.0 ± 0.0	0.0 ± 0.0	>100
**1l**	0.0 ± 0.0	6.3 ± 0.2	12.3 ± 0.3	>100

^a^ Values are mean ± SD (n = 3). The LD_50_ is one way to measure the short-term poisoning potential (acute toxicity) of a material.

#### 4.2.4 Molecular docking studies

**FIGURE 2:**
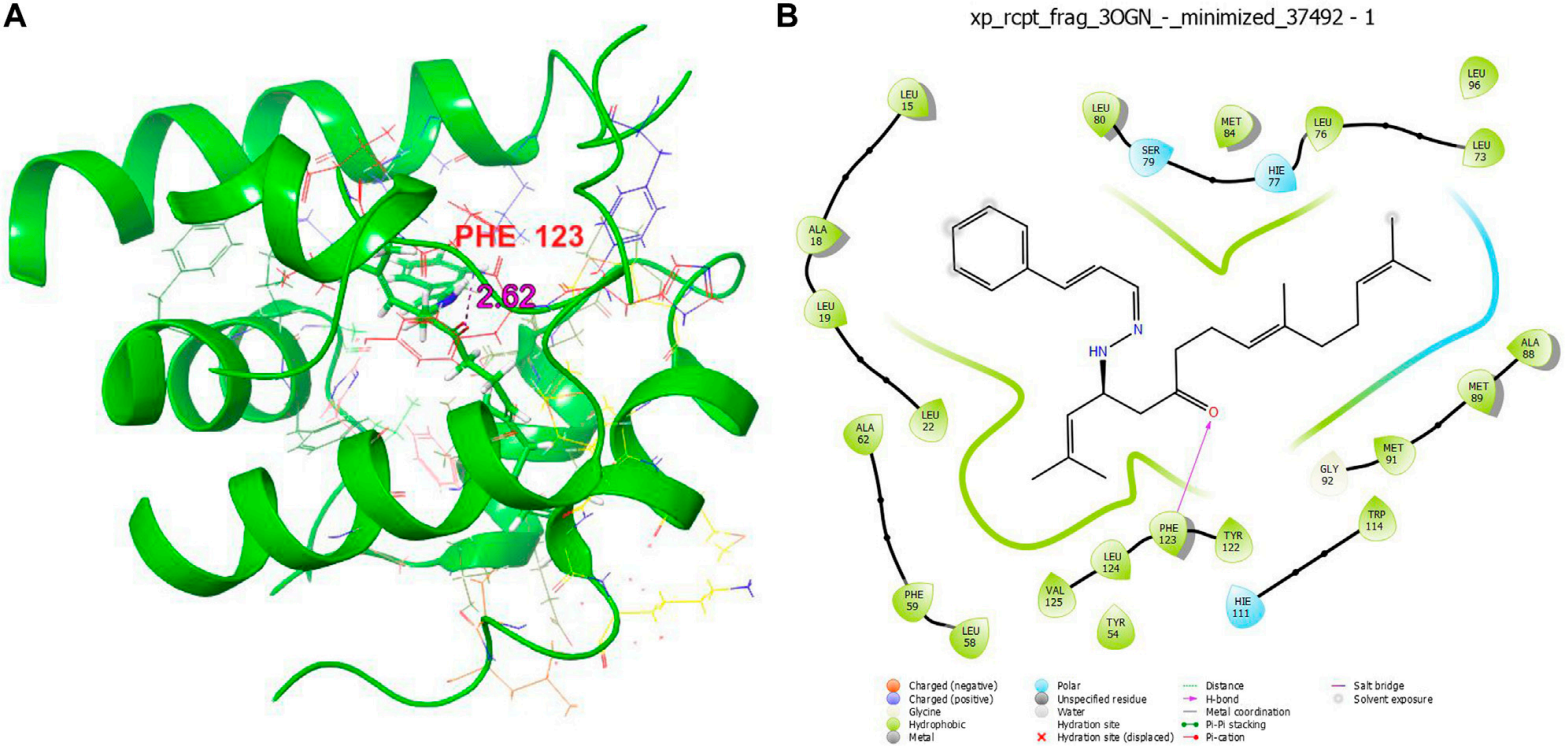
3D structure **(A)** and 2D structure **(B)** molecular docking studies of compound **1b**.

**FIGURE 3:**
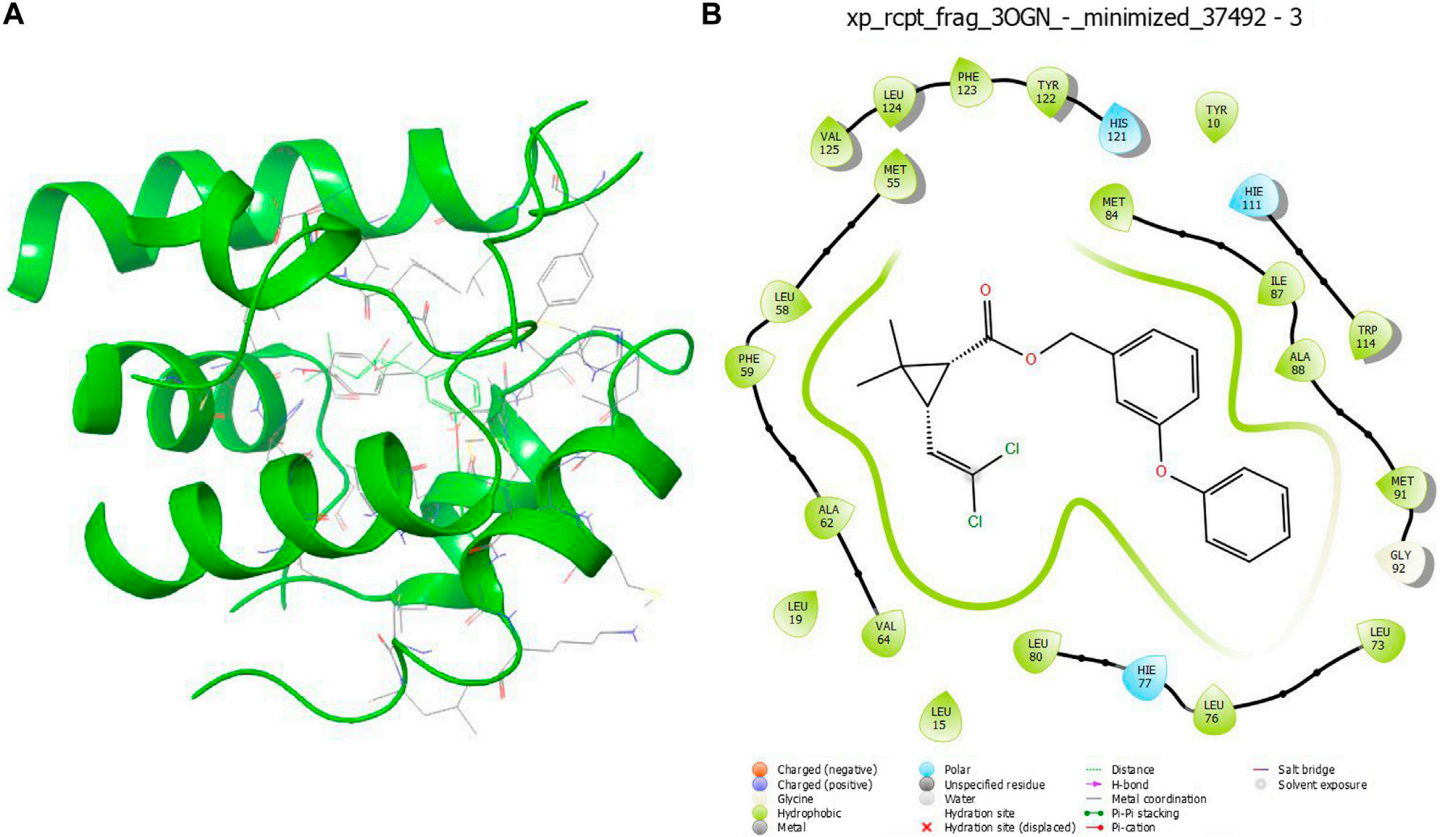
3D structure **(A)** and 2D structure **(B)** molecular docking studies of **permethrin**.

The compound **1b** and permethrin were analysis the protein that binds mosquito odourants. The amino acid residues that formed a pocket in the binding site were selected based on docking grid boxes. The AutoDock Vina software used to analyse the docking performance of compound **1b** and permethrin with 3OGN proteins. In (**1a-l**) receptors docked in 2D representation, the binding affinity of compound **1b** was significantly lower (−9.6 kcal/mol). Permethrin was used as a benchmark for its commercial availability in controlling mosquitoes. Stability protein-ligand interaction, which is formed when there is a suitable distance of less than 3.5 between the atoms of both the H-donor and H-acceptor. Compounds (**1a–l**), which formed potent hydrogen bonds with the corresponding 3OGN proteins with lengths less than 3.5 were discovered. Compound **1b** establishes hydrogen bond connections with the 3OGN receptor. With a hydrogen bond length of 2.62, Phe123 interacted with the hydrogen (Figure 2A). The amino acid residues Leu 15, Ala 18, Leu 19, Leu 22, Tyr 54, Leu 58, Phe 59, Ala 62, Leu 73, Leu 76, Hie 77, Ser 79, Leu 80, Met 84, Ala 88, Met 89, Met 91, Gly 92, Leu 96, Hie 111, Trp 114, Tyr 122, Phe 123, Leu 124, and Val 125 with a docking score of −9.6 kcal/mol (as presented in Table 7) suggest that the inhibitor molecule is involved in hydrophobic interactions, as shown in (Figure 2B). The result is that the interaction of the protein with the permethrin molecule (PDB ID: 3OGN) is depicted in permethrin, which is a medication. The amino acid residues Tyr 10, Leu 15, Leu 19, Met 55, Leu 58, Phe 59, Ala 62, Val 64, Leu 76, Hie 77, Leu 80, Met 84, Ile 87, Ala 88, Met 91, Gly 92, Hie 111, Trp 114, His 121, Tyr 122, Phe 123, Leu 124, and Val 125 (Figures 3A, B) interactions resulted in a docking score of (−10.5 kcal/mol) resulting in hydrophobic interactions (Table 7). A 2D image of the molecule docked with the receptor showed an inhibitory molecule situated within the binding pocket of the receptor, providing an overview of the molecular docking data.

**TABLE 7:** Docked result of **1b,** and permethrin (drug) with 3OGN using XP method.

S.No	Compound/Drug	Dock score	Interacting residues	Bond length
1	Permethrin (drug)	−10.5	-	-
2	1b	−9.6	Phe 123	2.62

##### 4.2.4.1 Structure-activity relationship

**FIGURE 4:**
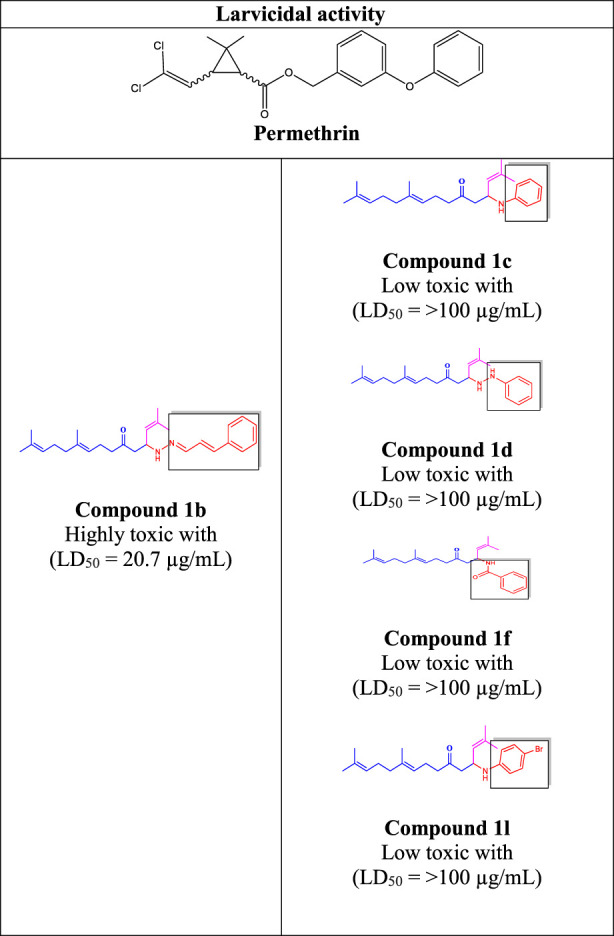
Structure-activity relationship.

The structure-activity relationship of the synthesised compounds (**1a-l**) were determined the structure-activity relationship is shown in (Figures 4). The maximum concentration used for analysis all compounds, among the synthesised compounds geranylacetone (42.3% mortality at 100 μg/mL, LD_50_: >100 μg/mL) due to presence of only acetone group, which very low active compared with other compounds, some substation added for compound **1i** (48.6% mortality at 100 μg/mL, LD_50_: 97.4 μg/mL) due to presence of (4-chlorobenzylidene)hydrazine with acetone give slightly increase the activity.

Compound **1d** (22.2% mortality at 100 μg/mL, LD_50_ value > 100 μg/mL) had no effect at all concentrations due to the presence of aniline with acetone, and compound **1l** (36.3% mortality at 100 μg/mL, LD_50_ value > 100 μg/mL) had no effect due to the presence of 4-bromo-aniline, both of which had very low activity compared with geranylacetone. The compound **1a** (80.2% mortality at 100 μg/mL, LD_50_ value 59.4 μg/mL), which have benzylidene-2-methylhydrazine with acetone group, **1e** (60.4% mortality at 100 μg/mL, (LD_50_: 66.0 μg/mL), dimethylaniline with acetone; **1h** (92.2% mortality at 100 μg/mL, LD_50_: 46.7 μg/mL) have *N,N*-dimethyl-4-((2-methylhydrazono)aniline with acetone, and **1j** (59.6% mortality at 100 μg/mL, (LD_50:_ 71.8 μg/mL) have (but-2-en-1-ylidene)hydrazine with acetone were moderately active compared with geranylacetone.

The compound **1b** (100% mortality at 100 μg/mL, LD_50_: 20.7 μg/mL) have 2-(3-phenyl allylidene)hydrazine contacting acetone, which shows that highly active whereas the compound **1g** (100% mortality at 100 μg/mL, LD_50_: 33.7 μg/mL) have (furan-2-ylmethy lene)-2-hydrazine contacting acetone, which shows that slightly low active compared with compound **1b**. Based on the structural activity relationship, the compound containing acetone connected to hydrazine was significantly highly active compared with other chemicals.

## 5 Conclusion

In this study, the catalytic role of the tyrosinase enzyme in the grindstone method was used to create the most efficient larvicidal active geranylacetone Mannich base derivatives. This process is affordable and produces a high yield. These substances have been studied for use as larvicides against *C. quinquefasciatus*. The larvicidal action of compound **1b** (−9.6 kcal/mol) and permethrin (−10.5 kcal/mol) are showed greater binding affinity for 3OGN. The compound **1b** (LD_50_: 20.7 μg/mL) was compared with permethrin (LD_50_: 24.4 μg/mL). Compound was identified as the most effective compound against *C. quinquefasciatus* out of all the compounds (**1a-l**) that were evaluated and the geranylacetone (LD_50_: >100 μg/mL) is low active compared with all compounds (**1a-l**). Compound **1b, 1d** and **1k** were identified with 0% mortality at 24 h against *O. mossambicus* in an antifeedant screening. Based on these observations of result findings, compound **1b** is the most effective larvicidal and non-toxic for non-target aquatic species in water. Therefore, active compounds may potentially function as a new class of larvicidal agents and constitute a promising foundation for the development of emerging ecologically significant bioactive compounds.

## Data Availability

The datasets presented in this study can be found in online repositories. The names of the repository/repositories and accession number(s) can be found in the article/Supplementary Material.
