# Supplementary material for: Mushroom tyrosinase enzyme catalysis: synthesis of larvicidal active geranylacetone derivatives against Culex quinquesfasciatus and molecular docking studies
Source: Front Chem. 2024 Jan 10;11:1303479. doi: 10.3389/fchem.2023.1303479 (PMC10806150; doi:10.3389/fchem.2023.1303479)
Supplement: Supplementary file 1 [file DataSheet1.pdf]

Mushroom Tyrosinase enzyme catalysis: Synthesis of Larvicidal Active Geranylacetone derivatives against *Culex Quinquesfasciatus* and Molecular Docking Studies

Janani Mullaivendhan[1], Anis Ahamed[2], Ibrahim A Arif[2], Gurusamy Raman[3], Idhayadhulla Akber[1*]

[1]Research Department of Chemistry, Nehru Memorial College (Affiliated Bharathidasan University), Puthanamapatti-621007, Tamil Nadu, India;

[2]Department of Botany and Microbiology, College of Science, King Saud University, P.O. Box 2455, Riyadh 11451, Saudi Arabia;

[3]Department of Life Science, Yeungnam University, Gyeongsan, Gyeongbuk-do, South Korea – 38541

Correspondence: a.idhayadhulla@gmail.com;

S.No.	Contents	Page no.
1	Figure 1-24: ^{1}H , and ^{13}C NMR spectrum of Compound **(1a-1i)**	2 -13

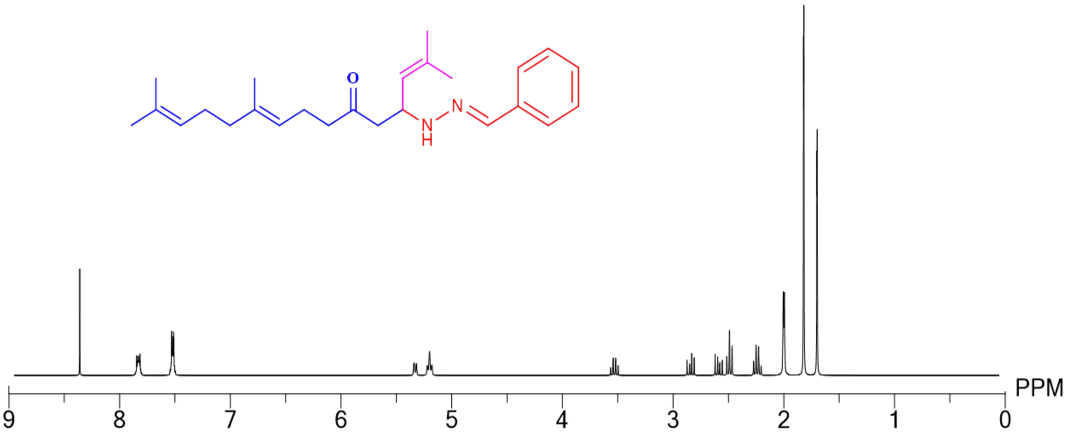

Figure 1. ^{1}H NMR spectrum of the compound **1a**

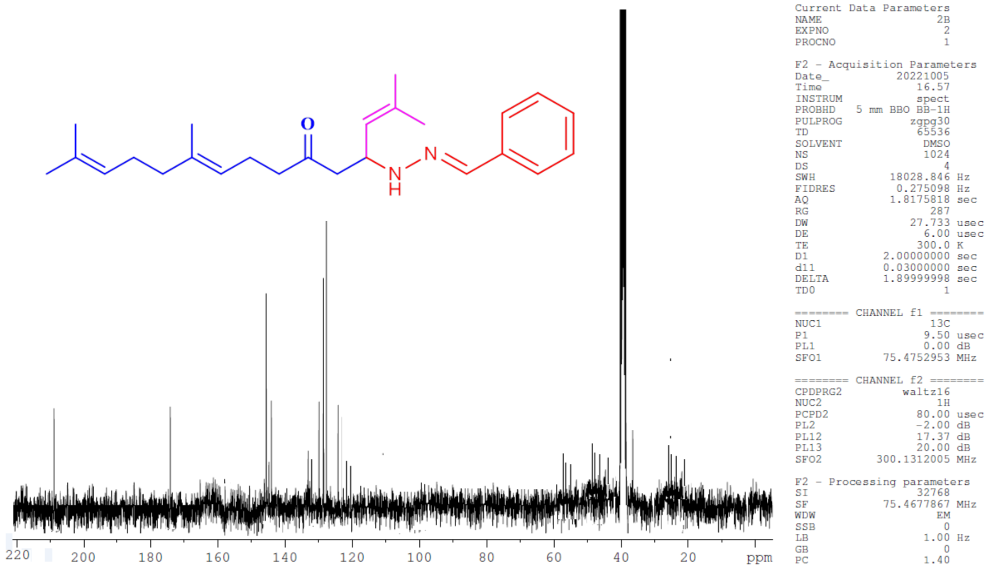

Figure 2. ^{13}C NMR spectrum of the compound **1a**

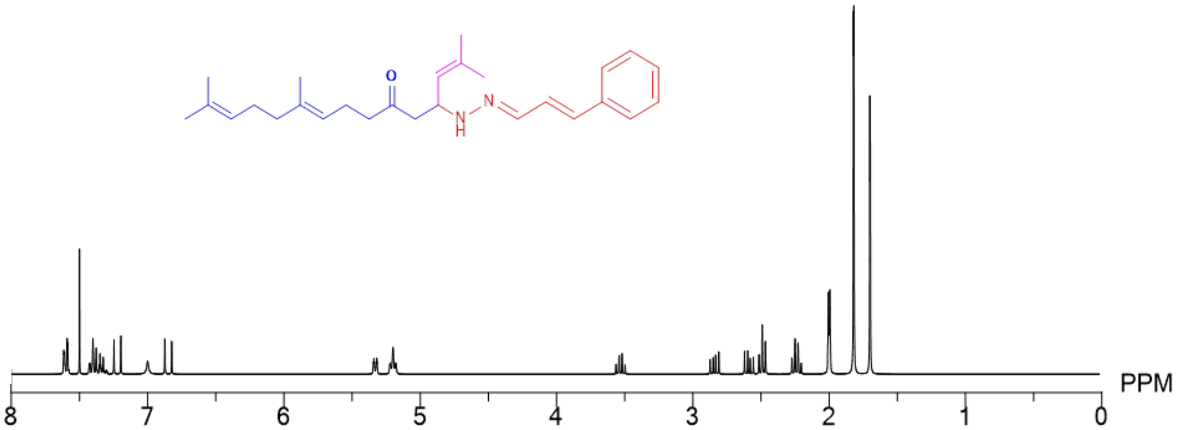

Figure 3. ^{1}H NMR spectrum of the compound **1b**

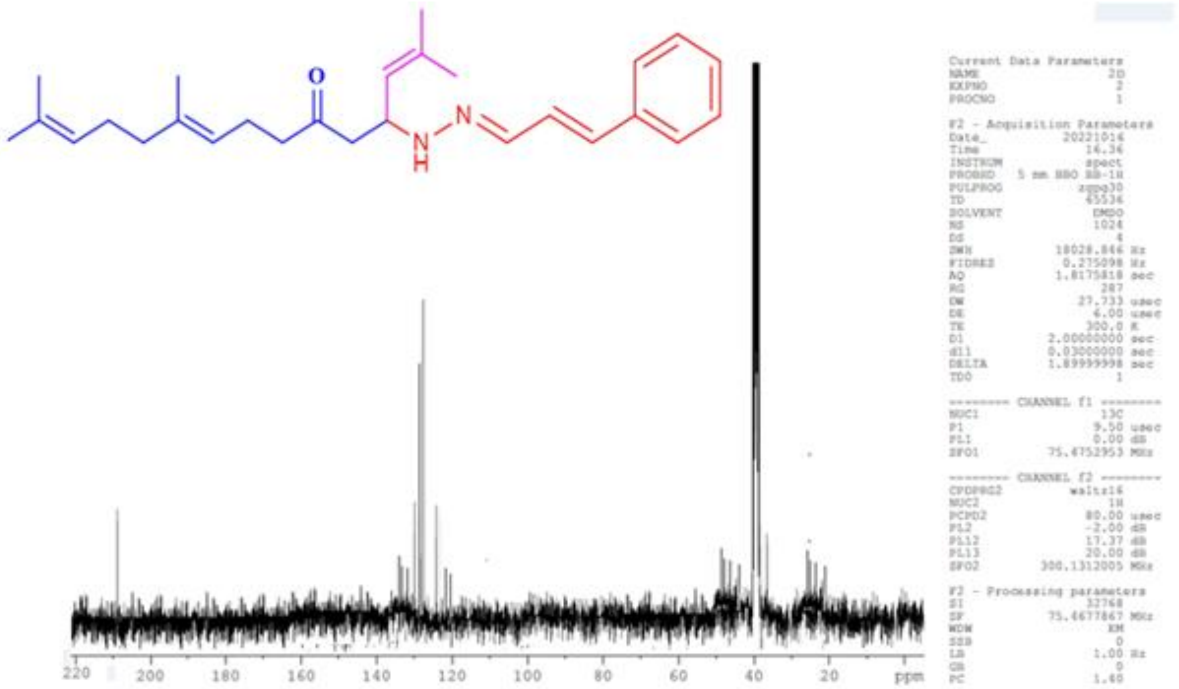

Figure 4.^{13}C NMR spectrum of the compound **1b**

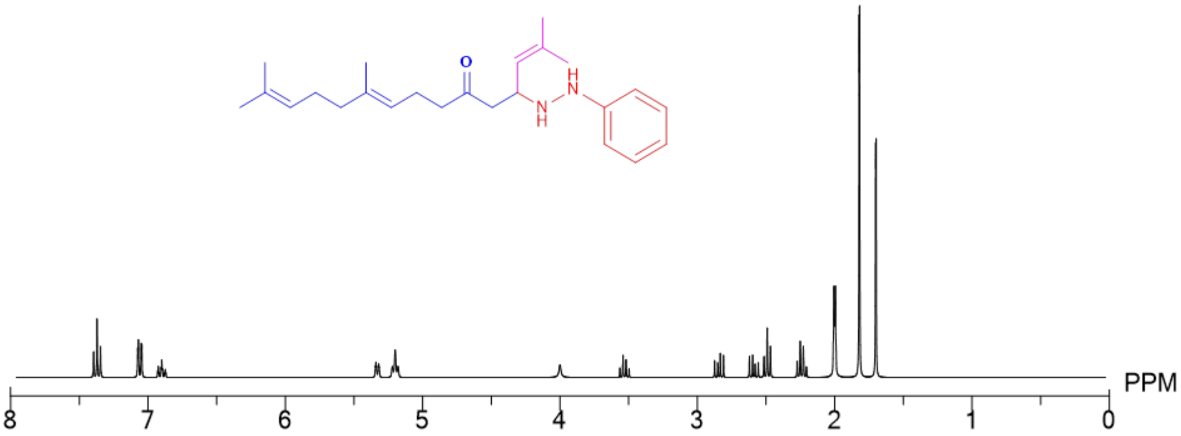

Figure 5. ^{1}H NMR spectrum of the compound **1c**

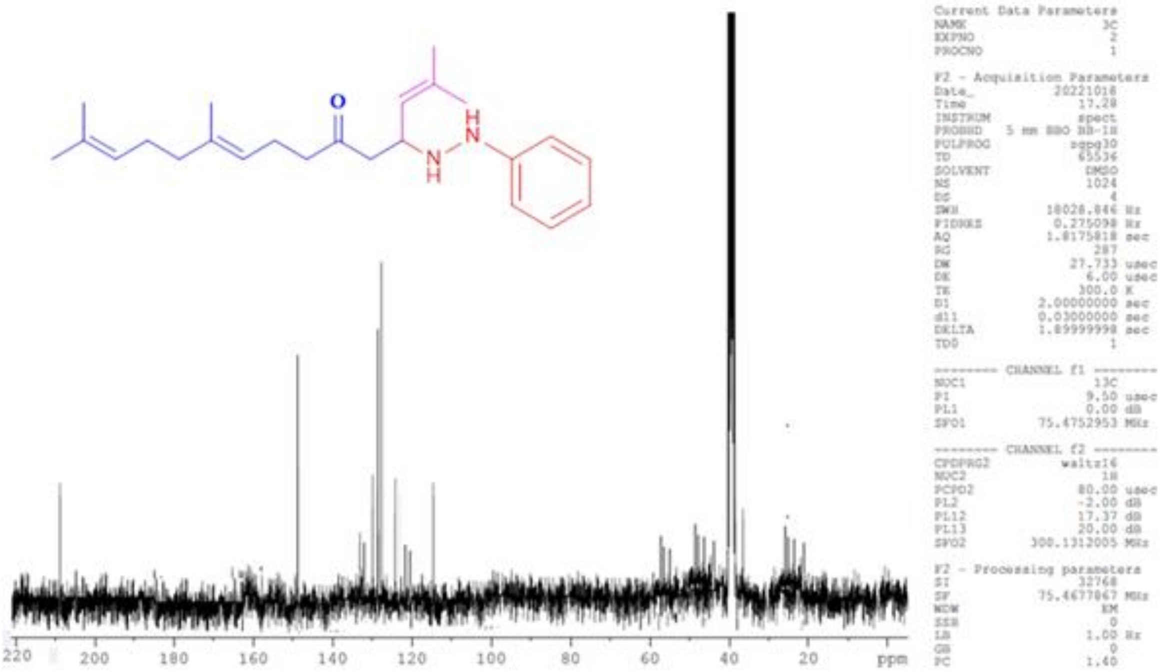

Figure 6.^{13}C NMR spectrum of the compound **1c**

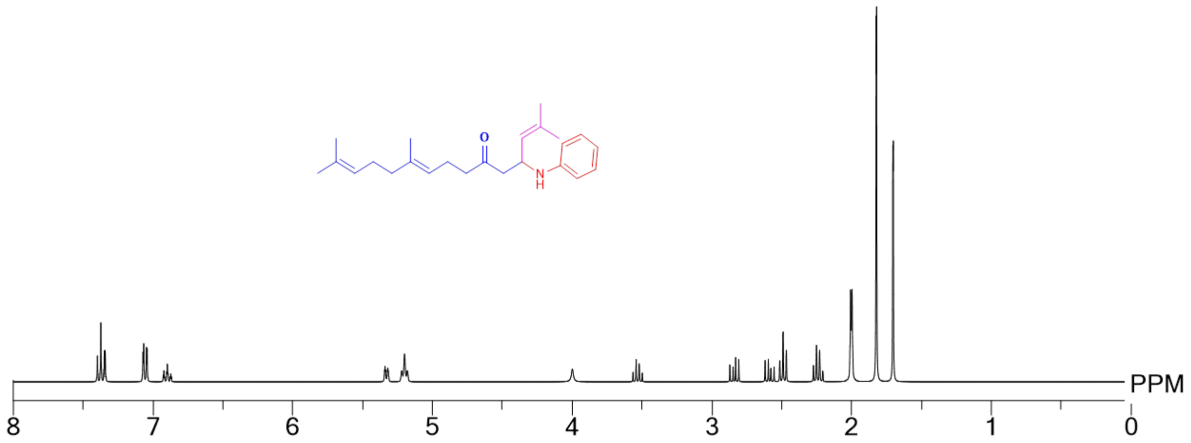

Figure 7. ^{1}H NMR spectrum of the compound **1d**

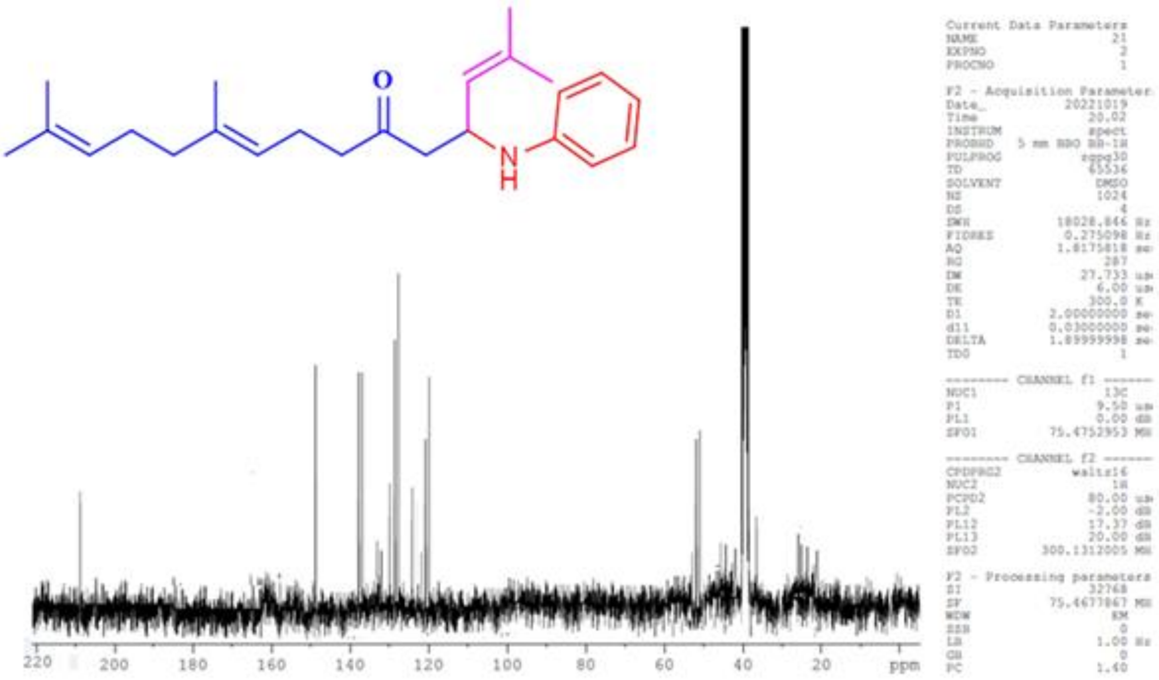

Figure 8.^{13}C NMR spectrum of the compound **1d**

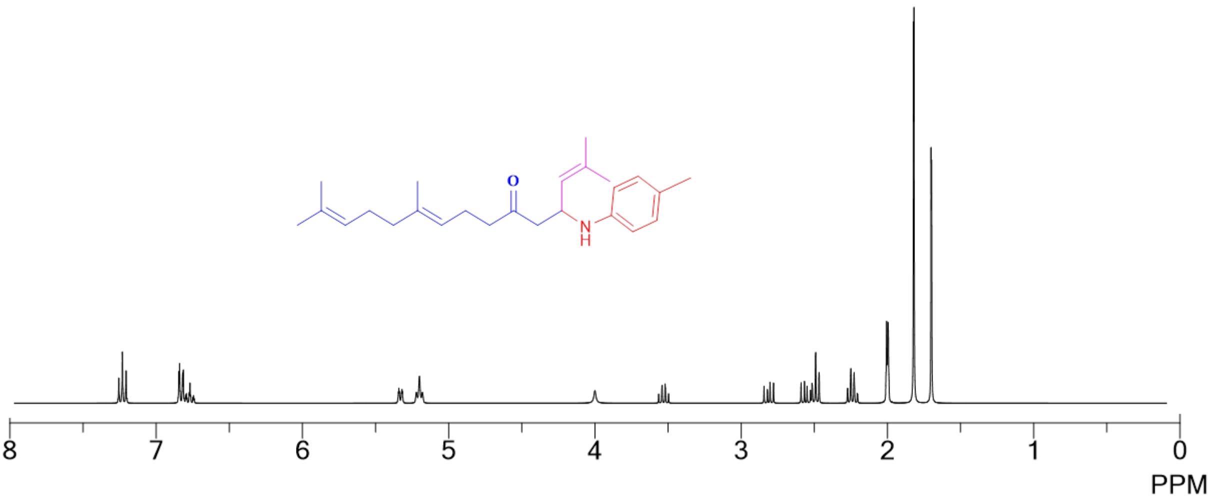

Figure 9. ^{1}H NMR spectrum of the compound **1e**

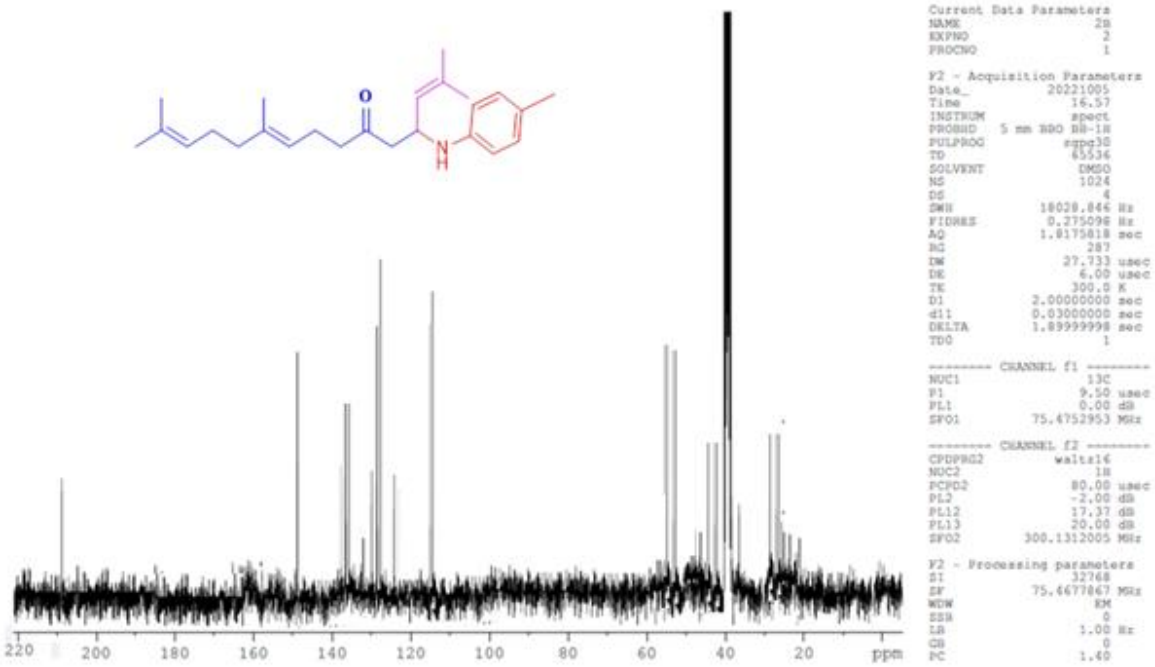

Figure 10. ^{13}C NMR spectrum of the compound **1e**

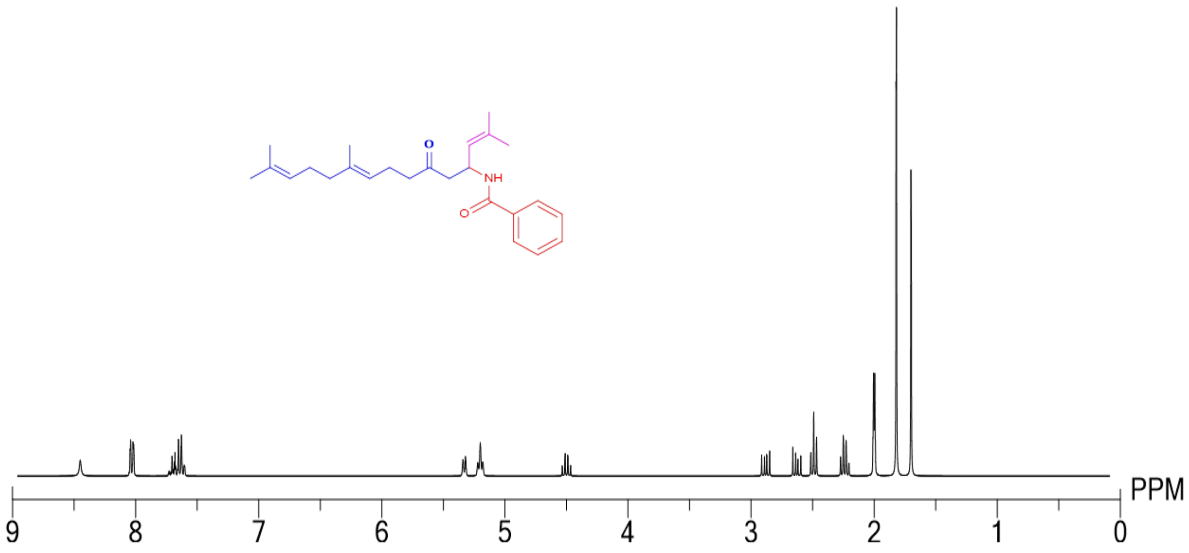

Figure 10. ^{1}H NMR spectrum of the compound **1f**

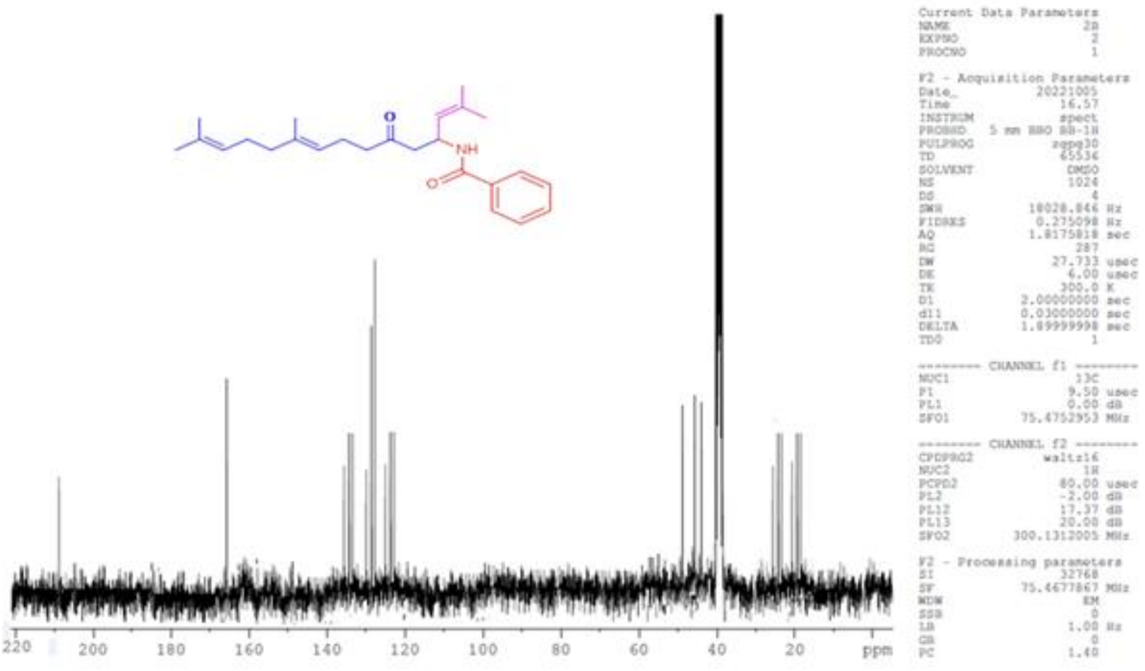

Figure 12.^{13}C NMR spectrum of the compound **1f**

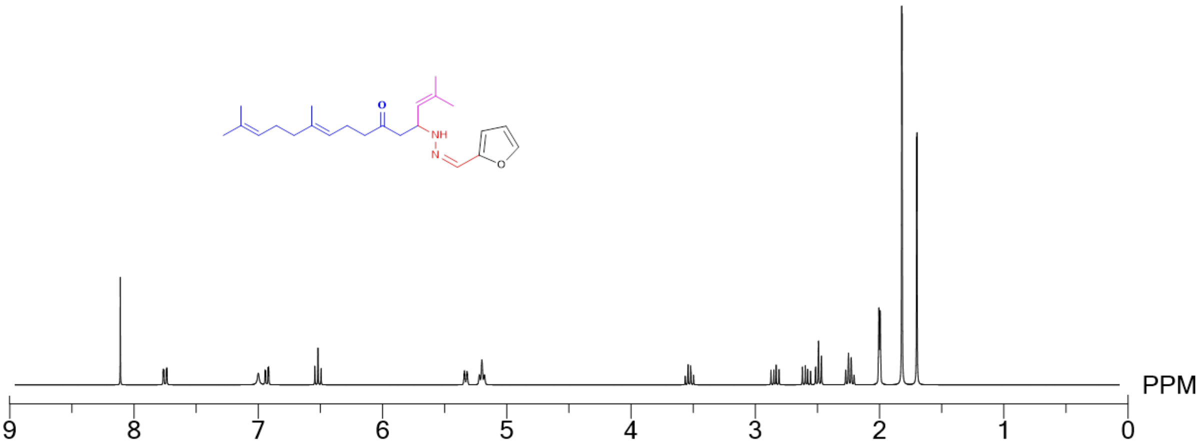

Figure 13. ^{1}H NMR spectrum of the compound **1g**

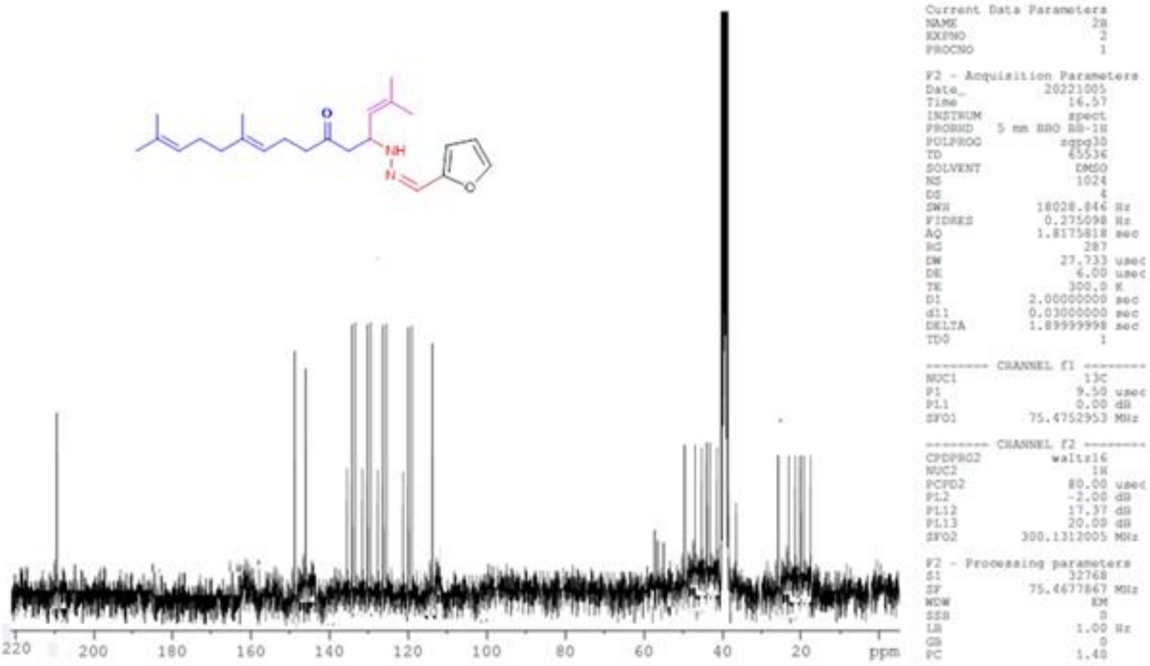

Figure 14.^{13}C NMR spectrum of the compound **1g**

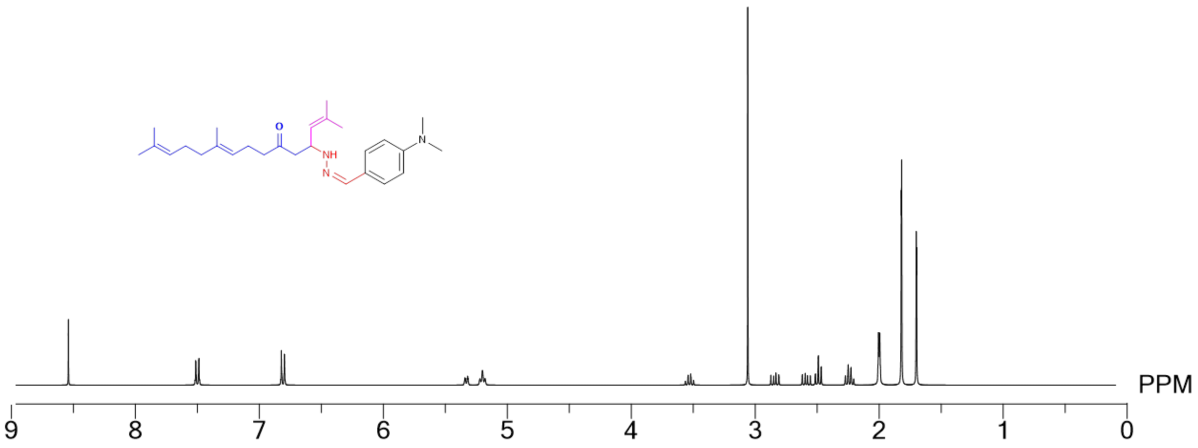

Figure 15. ^{1}H NMR spectrum of the compound **1h**

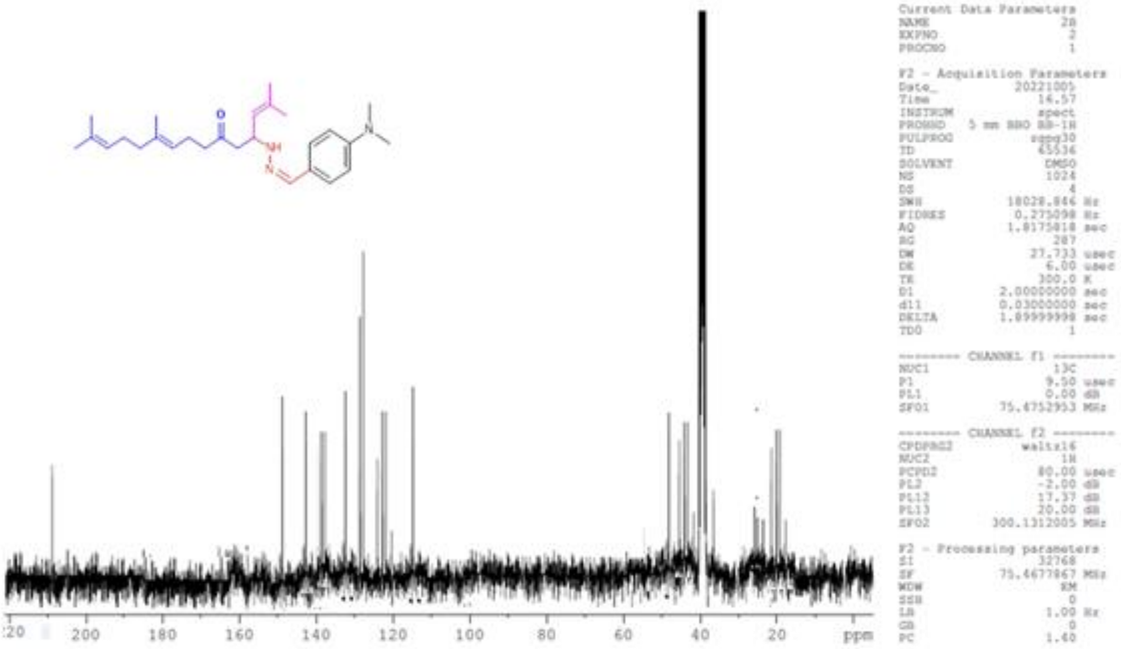

Figure 16.^{13}C NMR spectrum of the compound **1h**

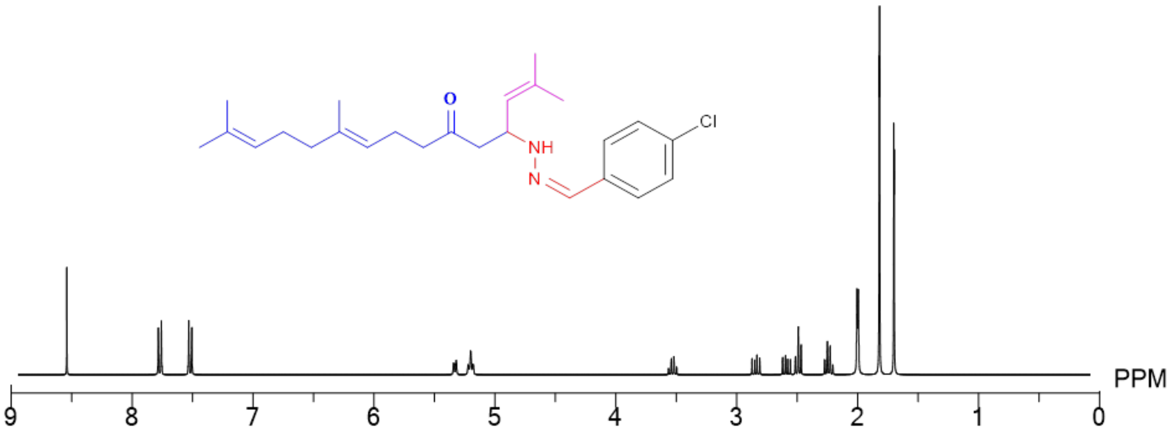

Figure 17. ^{1}H NMR spectrum of the compound **1i**

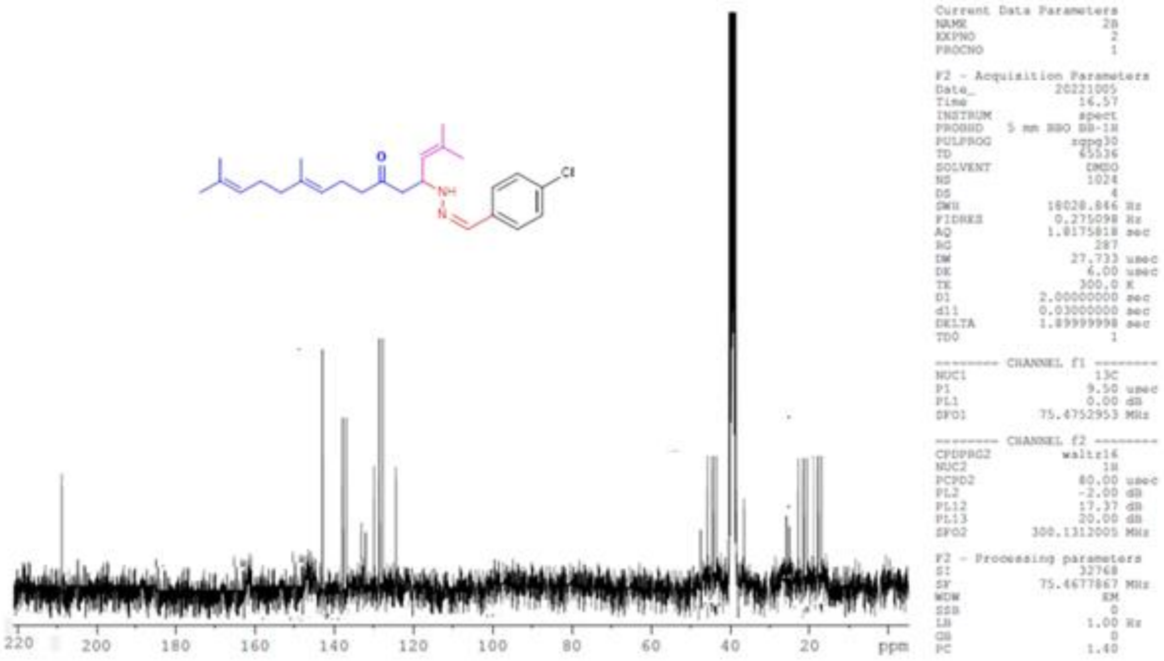

Figure 18.^{13}C NMR spectrum of the compound **1i**

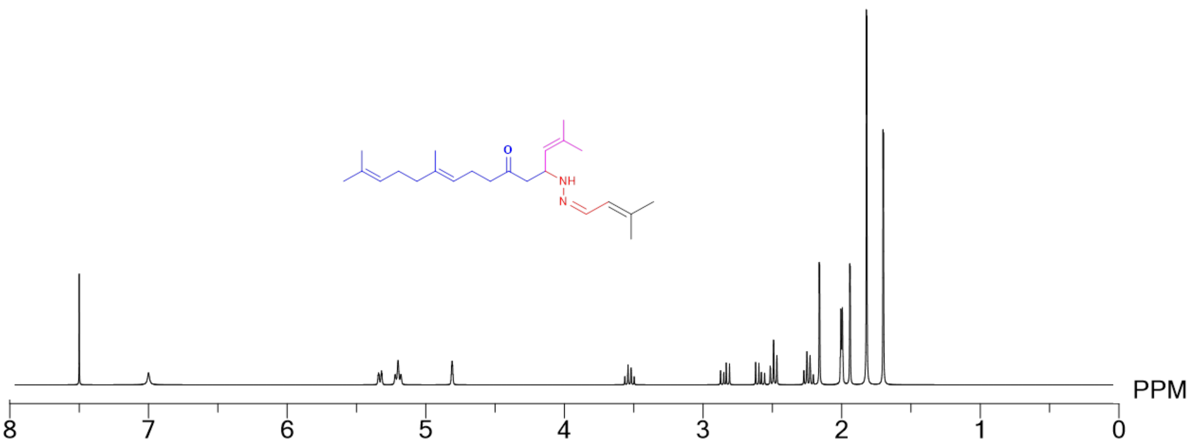

Figure 19. ^{1}H NMR spectrum of the compound **1j**

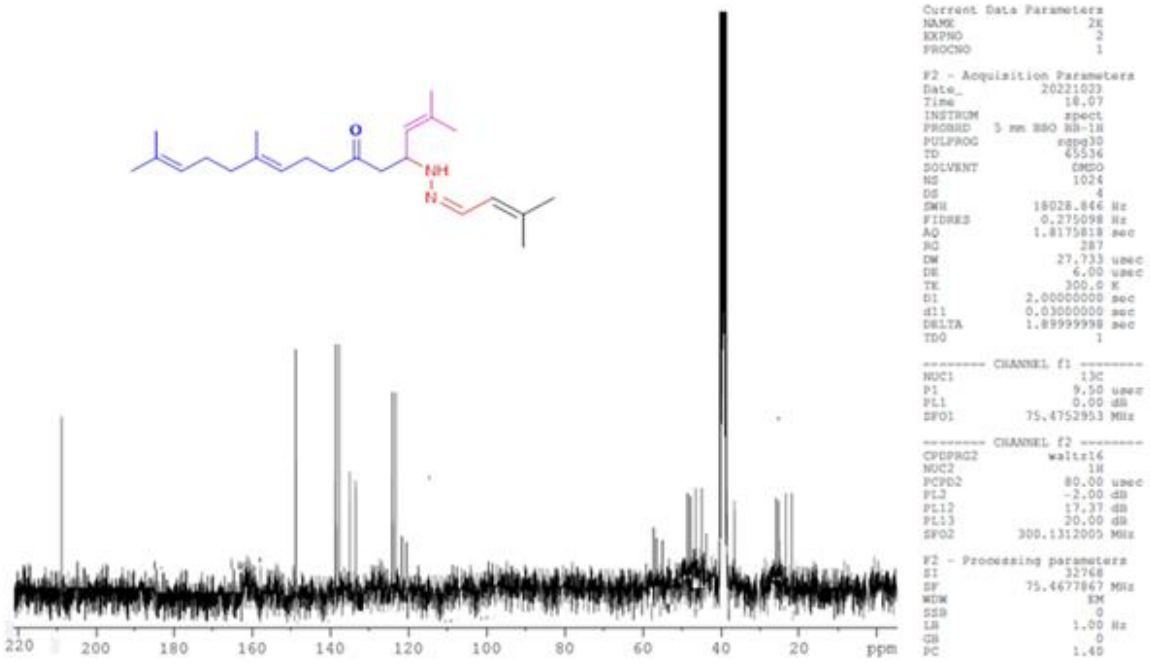

Figure 20. ^{13}C NMR spectrum of the compound **1j**

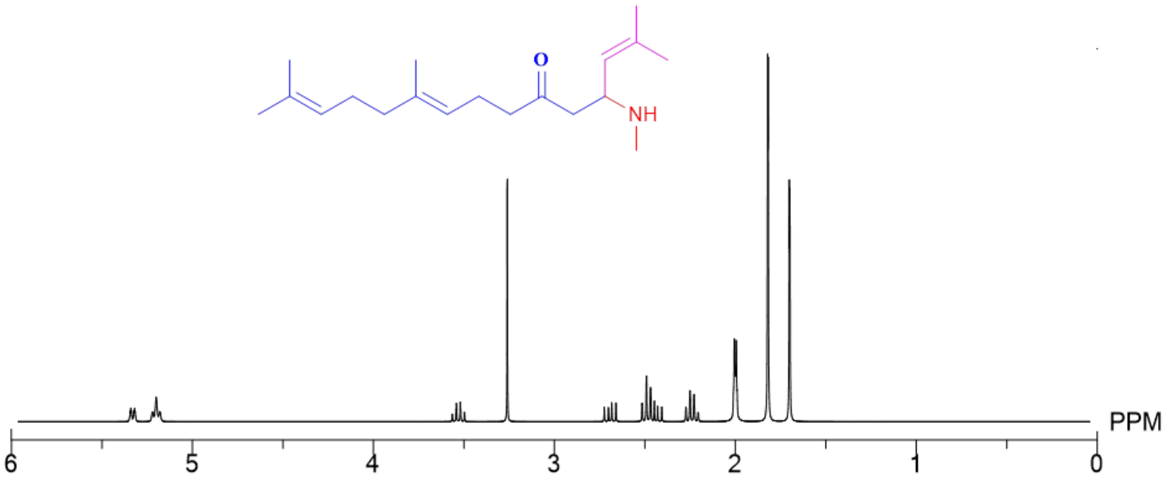

Figure 21. ^{1}H NMR spectrum of the compound **1h**

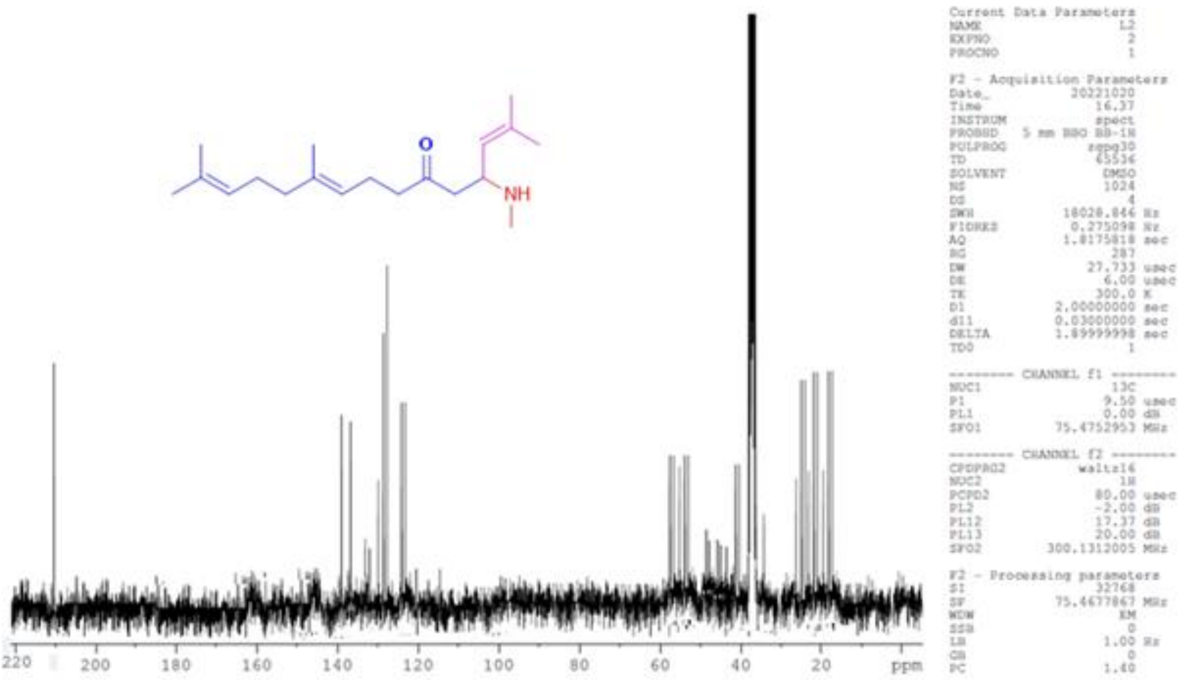

Figure 22. ^{13}C NMR spectrum of the compound **1h**

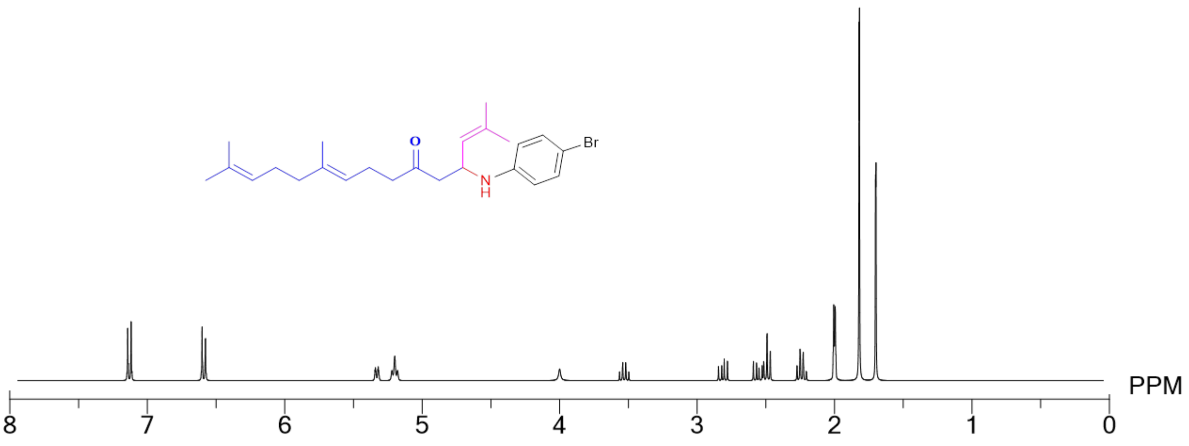

Figure 23. ^{1}H NMR spectrum of the compound **1i**

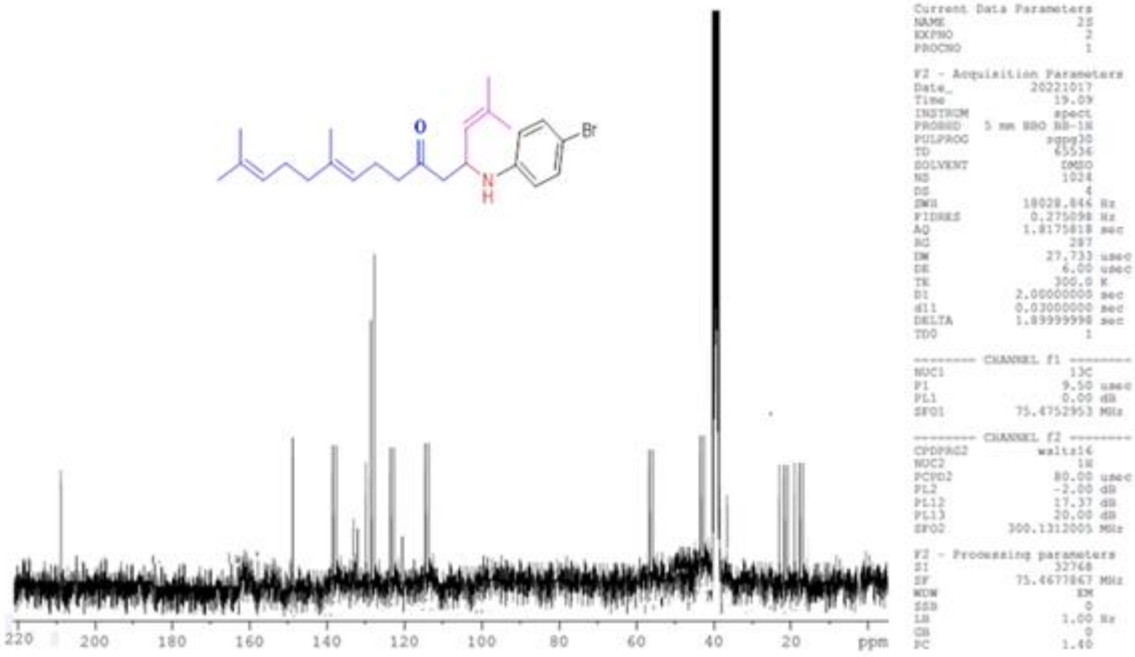

Figure 24.^{13}C NMR spectrum of the compound **1i**
